# MKK3 K329 Mutation Attenuates Diabetes-Associated Cognitive Dysfunction by Blocking the MKK3-RAGE Interaction and Inhibiting Neuroinflammation

**DOI:** 10.14336/AD.2024.0222

**Published:** 2024-02-22

**Authors:** Changjiang Ying, Yan Li, Shidi Wu, Lin Gao, Yandong Zhu, Ye Qian, Xiangru Wen, Hui Li, Chengyu Huang, Bin Hu, Yuanjian Song, Xiaoyan Zhou

**Affiliations:** ^1^Xuzhou Engineering Research Center of Medical Genetics and Transformation, Department of Genetics, Xuzhou Medical University, Xuzhou, Jiangsu, China.; ^2^Department of Endocrinology, Affiliated Hospital of Xuzhou Medical University, Xuzhou, Jiangsu 221002, China.; ^3^The Graduate School, Xuzhou Medical University, Xuzhou, Jiangsu 221004, China.; ^4^Jiangsu Key Laboratory of Brain Disease and Bioinformation, Research Center for Biochemistry and Molecular Biology, Xuzhou Medical University, Xuzhou, China.

**Keywords:** Diabetes-associated cognitive dysfunction, Neuroinflammation, Mitogen-activated protein kinase kinase3, Receptor for advanced glycation end products, Nuclear factor-κB;

## Abstract

The receptor for advanced glycation end products (RAGE) contributes to diabetes-associated cognitive dysfunction (DACD) through the interaction of its C-terminal AAs 2-5 with mitogen-activated protein kinase kinase 3 (MKK3). However, the associated MKK3 binding site is unknown. Here, db/db mice were used as a model for type 2 diabetes. GST pull-down assays and AutoDock Vina simulations were conducted to identify the key RAGE binding site in MKK3. This binding site was mutated to investigate its effects on DACD and to elucidate the underlying mechanisms. The interaction of MKK3 and RAGE, the levels of inflammatory factors, and the activation of microglia and astrocytes were tested. Synaptic morphology and plasticity in hippocampal neurons were assessed via electrophysiological recordings and Golgi staining. Behavioral tests were used to assess cognitive function. In this study, MKK3 bound directly to RAGE via its lysine 329 (K329), leading to the activation of the nuclear factor-κB (NF-κB) signaling pathway, which in turn triggered neuroinflammation and synaptic dysfunction, and ultimately contributed to DACD. MKK3 mutation at K329 reversed synaptic dysfunction and cognitive deficits by downregulating the NF-κB signaling pathway and inhibiting neuroinflammation. These results confirm that neuroinflammation and synaptic dysfunction in the hippocampus rely on the direct binding of MKK3 and RAGE. We conclude that MKK3 K329 binding to C-terminal RAGE (ct-RAGE) is a key mechanism by which neuroinflammation and synaptic dysfunction are induced in the hippocampus. This study presents a novel mechanism for DACD and proposes a novel therapeutic avenue for neuroprotection in DACD.

## INTRODUCTION

Type 2 diabetes mellitus (T2DM) is a prevalent chronic metabolic disease characterized by persistent hyperglycemia, which exerts detrimental effects on multiple vital organs and predisposes individuals to cardiovascular diseases, neuropathy, and kidney disease [1]. It is estimated that by 2045, T2DM will affect about 693 million people globally. T2DM is thus a major public health concern, placing a heavy burden on healthcare resources and hampering economic development [2]. Epidemiological studies have shown that diabetes is also an independent risk factor for cognitive impairment, and individuals with diabetes have an increased risk (1.6-fold) of developing Alzheimer's disease [3]. With the rising number of diabetic patients, diabetes-associated cognitive dysfunction (DACD) has become a focus of research [3,4]. To prevent and treat DACD, it is essential to understand the underlying mechanisms.

DACD is a transitional stage between normal cognition and Alzheimer’s disease, which is characterized by impairments in processing speed, attention, executive function, and memory [5]. Recent studies have shown that these cognitive deficits are accompanied by changes in brain signal conduction, neuronal apoptosis, loss of dendritic complexity, a reduced number of synapses, and decreased plasticity, which are all associated with neuroinflammation [6,7]. Mitogen-activated protein kinase kinase 3 (MKK3) is a dual threonine/tyrosine protein kinase that regulates inflammation, proliferation, and apoptosis through specific phosphorylation and activation of the p38 mitogen-activated protein kinase (MAPK) [8,9]. As a component of the innate immune response pathway, MKK3 is required for inflammatory cell recruitment and accelerates disease progression and complications in T2DM [10]. Of note, MKK3 regulates the p38 MAPK pathway in hippocampal neurons, impairing their function and ultimately resulting in diabetic encephalopathy [11]. Despite these recent advances in understanding, further investigation is required to establish whether and how MKK3 contributes directly to diabetes-related neuroinflammation.

Advanced glycation end products (AGEs) generated by hyperglycemia are a complex and heterogeneous group of compounds known to be involved in diabetes-related neuropathology [12]. The interaction between AGEs and its receptor RAGE controls the secretion of inflammatory cytokines, thereby promoting hyperglycemia-induced neuronal damage and accelerating cognitive decline [13]. The AGE-RAGE pathway activates several types of intracellular signaling; neuroinflammation relies mainly on the cytoplasmic domain of RAGE [14]. Our previous study demonstrated that the direct binding of RAGE to MKK3 in hippocampal neurons worsened cognitive function in db/db mice, and that C-terminal AAs 2-5 of RAGE are the crucial sites for its interaction with MKK3 [11]. However, the corresponding key sites in MKK3 have yet to be unidentified. In this study, we therefore focused on deepening our understanding of the contribution of MKK3 to neuroinflammation to shed light on the cellular and molecular mechanisms that underlie DACD.

Here, we report that MKK3 binds to RAGE via its lysine 329 (K329). In db/db mice, this interaction was significantly correlated with a decline in cognition, as well as the hippocampal neuroinflammation and synaptic dysfunction. Disrupting the interaction between MKK3 and RAGE with MKK3 mutation could improve cognitive function through dampening the activation of the NF-κB signaling pathway and then inhibiting neuroinflammation. This study demonstrates for the first time that MKK3 participates in DACD and elucidates the underlying molecular mechanisms. Targeting MKK3 may improve therapeutic strategies for diabetes-related neurodegenerative diseases.

## METHODS AND MATERIALS

### Antibodies and reagents

The primary antibodies used in this study for western blotting, Co-immunoprecipitation, and immune-fluorescence were as follows: Mouse Anti-RAGE (Santa Cruz Biotechnology, sc-365154), Rabbit Anti-MKK3 (Cell Signaling Technology, 8535), Mouse Anti-Caspase-1 (Adipogen, AG-20B-004), Rabbit Anti-IL-1β (Proteintech, 16806-1-AP), Rabbit Anti-TNF-α (Cell Signaling Technology, 119485), Mouse Anti-GST (Proteintech, 10000-0-AP), Rabbit Anti-His (Proteintech, 66005-1-Ig), Mouse Anti-IgG (Proteintech, B9006 20), Rabbit Anti-IgG (Proteintech, 10285-1-AP), Mouse Anti-β-Actin (Cell Signaling Technology, 3700), Rabbit Anti-LaminB (Proteintech, 12987-1-AP), Rabbit Anti-p65 (Cell Signaling Technology, 8242), Mouse Anti-P-p65 (Proteintech, 82335-1-RR), Rabbit Anti-p50 (Proteintech, 15506-1-AP), Rabbit Anti-IBA-1 (Wako, 019-19741) and Mouse Anti-GFAP (Cell Signaling Technology, 3670). Secondary antibodies for western blotting, Co-immunoprecipitation and immunofluorescence were included Rabbit Anti-IgG (VICMED, V926-32211), Mouse Anti-IgG (VICMED, V926-32210), Goat anti-rabbit Alexa Fluor 488 (Proteintech, VA1022) and Goat anti-mouse Alexa Fluor 594 (Proteintech, VA1023). All chemicals, recombinant proteins, critical commercial assays, cell lines, experimental models, oligonucleotides, and recombinant DNA used in the present work were presented in Supplementary Table 1.

### Cell culture: experimental design

HT-22 cells were cultured in Dulbecco's modified Eagle's medium (DMEM) with 10% fetal bovine serum (FBS) and 1% penicillin/streptomycin and were maintained at 37°C with 20% O_2_ and 5% CO_2_. Cells were randomly divided into five groups: a normal-glucose group (NG; 25 mM glucose); a high-glucose group (HG; 50 mM glucose); a high-glucose plus RAGE-inhibitor group (FPS; 50 mM glucose treated with 2.5 μM FPS-ZM1 dissolved in DMSO); a solvent control group (DMSO; 50 mM glucose treated with 2.5 μM DMSO), and a mannitol control group (MG; an isosmotic pressure control, 25 mM glucose treated with 25 mM mannitol). After synchronization, cells in the HG, FPS, and DMSO groups were subjected to high glucose for 24 h and then collected all cells for further experimentation.

In the second experiment, MKK3 expression in HT-22 cells was reduced by transfecting the cells with lentivirus (LV)-MKK3-shRNA. Two weeks later, stable MKK3-knockdown cells were divided into four groups: a high-glucose group (HG+LV-MKK3-shRNA); a group that received His-labeled wild-type MKK3, to overexpress wild-type MKK3, followed by high-glucose treatment (HG+LV-MKK3-shRNA+WT); a group that received His-labeled mutated MKK3, to overexpress MKK3 with a mutation at K329, followed by high-glucose treatment (HG+LV-MKK3-shRNA+Mut); and a group that received His-labeled MKK3 transfected with a nonsense control followed by high-glucose treatment (HG+LV-MKK3-shRNA+NC). Cells were collected 24 h after high-glucose stimulation.

### Mice: experimental design

Male db/db mice (BKS.Cg-m^+/+^ Leprdb/J) and their age- and sex-matched normoglycemic heterozygous littermate db/m (BKS.Cg-m^+/+^ Leprdb^/+/J^) controls were obtained from Jackson Laboratory. Eight-week-old db/db mice were allocated into five groups: db/db mice that received no additional treatment (db/db); db/db mice that received bilateral microinjection of LV-MKK3-shRNA in the hippocampus to knock down MKK3 (db/db+LV-MKK3-shRNA); db/db mice that received LV-MKK3-shRNA injections followed two weeks later by injection of wild-type MKK3 (db/db+LV-MKK3-shRNA+WT); db/db mice that received LV-MKK3-shRNA injections followed two weeks later by injection of mutant MKK3 (db/db+LV-MKK3-shRNA+Mut), and db/db mice that received LV-MKK3-shRNA injections followed two weeks later by injection of a nonsense control (db/db+LV-MKK3-shRNA+NC). Age- and sex-matched db/m mice served as an additional control group (db/m). At the age of 14 weeks, mice were sacrificed for further study.

All mice were maintained under specific pathogen-free conditions and housed in clear plastic cages on a 12 h/12 h light/dark cycle at 23±1°C with free access to water and food. Animal experimental procedures were conducted by the guidelines described in the revised 2011. Chinese Regulations for the Administration of Affairs Concerning Experimental Animals and approved by the Institutional Animal Care and Use Committee of Xuzhou Medical University (protocol: 202207S074).

### Cell transfection

HEK-293T cells were used after 4 to 5 passages. Briefly, pCDNA3.1-MKK3-His or different pCDNA3.1-MKK3-His mutants were transfected into cells with the EL transfection reagent according to the manufacturer’s protocol. 24 h later, cells were collected, and proteins were extracted for further experimentation.

HT-22 cells were first transfected with LV-MKK3-shRNA to knock down MKK3. After two weeks, stable knockdown cells were transfected with RFP-labeled LV-MKK3-His-WT/Mut (to overexpress either wild-type MKK3 or MKK3 with a K329A mutation). The medium was replaced with fresh complete medium 24 h after transfection. Then, the medium was replaced each day with a medium containing puromycin (6 μg/mL) or neomycin (400 μg/mL) to eliminate uninfected cells. Two weeks later, stable cells were stimulated with high glucose.

### Flow cytometry analysis

The apoptosis rate was determined using the Annexin V-APC Apoptosis Detection kit [15]. Briefly, cells were collected with trypsin without EDTA, and 1×106/100 μL of cells were placed in a flow tube washed and suspended in PBS three times. Then, 500 μL of binding buffer and 5 μL of Annexin V-APC were added to each flow tube, which was then placed in a dark room for 15 min. The apoptosis rate was detected by flow cytometry, and the data were processed using FlowJo software.

### Intra-hippocampal injections

Mice (8 weeks old) were anesthetized with 1.5% pentobarbital (0.6 mL/g) and then fixed in a stereotaxic frame. The hippocampal CA1 subregion was targeted bilaterally with stereotaxic coordinates of AP -2.0 mm, ML ±1.7 mm, and DV -2.2 mm [11]. LV-MKK3-shRNA was injected at a speed of 0.25 μL/min, and the microinjector was kept in place for 5 min after the injection. In some groups of mice, to overexpress wild-type or mutant MKK3 in knockdown mice, EGFP-tagged LV-MKK3-His-Wt or -Mut under the neuronal CAP-Syn promoter was injected into the bilateral hippocampus two weeks later using the same procedure. After 4 weeks of recovery and viral expression, further experiments were conducted.

### Sample preparation

For cellular protein, cells were washed three times with cold PBS and collected via centrifugation at 120 *g* for 10 min. For animal protein, mice were anesthetized then the hippocampus was dissected carefully on ice, frozen rapidly in liquid nitrogen, and stored at -80°C until use. Nuclear and cytoplasmic proteins were extracted with special protein extraction kits. Briefly, the protein sample was mixed with three volumes of cold homogenization buffer A containing phosphatase inhibitor cocktails and phenylmethanesulfonyl fluoride (PMSF). The mixture was centrifuged at 1200 *g* for 5 min and then the supernatant was harvested as the cytosolic fraction. The precipitate was resuspended with five volumes of cold homogenization buffer B and centrifuged at 1500 *g* for 10 min, then the supernatant was collected as the nuclear fraction. Proteins were quantified with a BCA Protein Assay Kit and the concentrations were counted according to the Lowry method.

### Western blotting

Equal amounts of proteins were separated by SDS-PAGE and then electro-transferred onto the nitrocellulose (NC) membrane [15]. After blocking, the membranes were incubated overnight at 4°C with the indicated primary antibodies diluted in a blocking buffer. After washing three times with washing buffer, the NC membranes were incubated with the corresponding secondary antibodies for 2 h at room temperature. The stamps in the immunoreactive bands were scanned with an Odyssey Laser imaging scanner and analyzed in ImageJ.

### Co-immunoprecipitation

Proteins (200 μg) were diluted with 400 μL immunoprecipitation buffer. Co-IP was conducted by incubating with the antibodies or control IgG overnight at 4°C followed by adding protein A/G-Agarose for 2 h at 4°C. The mixture was then washed five times by centrifugation at 1000 *g* for 2 min at 4°C with immunoprecipitation buffer. Finally, the supernatant was eluted by the addition of sample volume buffer and then subjected to western blot analysis [16].

### Immunofluorescence

To evaluate the co-localization of MKK3 and RAGE, HT-22 cells were fixed with 4% paraformaldehyde and permeabilized with PBS containing 0.3% Triton X-100 for 30 min. Cells were incubated with MKK3 and RAGE primary antibodies overnight at 4°C and blocked the next day with 1% goat serum for 1 h. The following day, after washing three times with 1% PBS, cells were incubated with Alexa Fluor 488-conjugated rabbit or Alexa Fluor 594-conjugated mouse secondary antibodies for 2 h followed by washing three times and dying with DAPI for 8 min. Images were surveyed with a confocal microscope (Zeiss LSM710), and the overlap value (the percentage of colocalization of red fluorescence and green fluorescence) was calculated by the Zeiss system software. All immunostaining procedures included a control with only the secondary antibody to distinguish genuine target staining from the background [17].

For IBA-1 and GFAP immunohistochemistry, mice were deeply anesthetized and then intracardially perfused with saline solution and 4% paraformaldehyde in 0.1 M phosphatebuffer (PB). Brains were removed immediately, fixed in 4% paraformaldehyde overnight, and equilibrated in 30% sucrose at 4°C. Twenty- micrometer thick serial coronal sections were taken on a freezing microtome. Nine sections from each group were selected for study (two or three brain sections per mouse). After rinsing with 0.1 M PBS (30 min) and blocking with 0.2% (vol/vol) Triton X-100 and 10% (wt/vol) normal goat serum in 0.1 M PBS for 1 h, sections were incubated with the appropriate primary antibodies (IBA-1 and GFAP) overnight at 4°C. The next day, sections were incubated with Alexa Fluor 488 or Alexa Fluor 594-conjugated secondary fluorescent antibodies respectively in a dark room. Nuclei were stained with DAPI for 10 min at room temperature. Confocal images were captured by a Zeiss LSM710 confocal microscope. DAPI and IBA-1 or GFAP-positive cells were considered to be activated microglia or astrocytes. The number of activated microglia or astrocytes was calculated per 1-mm length of the hippocampal CA1 subregion.

### Docking methods

An MKK3 model was built with Swiss-Model (https://swissmodel.expasy.org/) and the C-terminal of RAGE (ct-RAGE) was obtained from the Protein Data Bank database (https://www.rcsb.org/structure/2LMB). Docking experiments were conducted in AutoDock Vina (version 1.1.2) [11]. Amino acids in the C-terminal of RAGE were set as flexible residues and, except for peptide bonds, all bonds could rotate. A genetic algorithm was used to minimize system energy and prepare docking conformations using the default settings, and about 50 possible substrate conformations were established by docking. The conformations with the highest docking scores are displayed and were used for analyses. All model Figures representing protein interactions were created in PyMOL (version 0.99).

### GST pull-down

The His-tagged vector PcDNA3.1 was used to overexpress MKK3 and four mutations (Q104A, H203A, K205A, and K329A), and was transfected into HEK-293T cells. The overexpressed proteins were lysed in a homogenization buffer after overexpression. The GST-tagged vector pGEX-4T-1 was used to express RAGE and was translated and overexpressed in BL21 cells, followed by protein purification according to a GST protein interaction pull-down kit, following the manufacturer's instructions [18]. Briefly, 50 μL TBS containing the pull-down lysis buffer to pre-equilibrated agarose resin, and then 200 μL GST-tagged RAGE was co-incubated with the resin at 4°C for 6-8 h on a rotating platform, followed by washing five times. Wild-type or mutated MKK3 overexpressed protein (200 μL) was added to the GST-tagged protein for 2 h. Finally, the protein interaction was assessed by western blotting.

### Electrophysiological recordings

Electrophysiological recordings were conducted following the previously described method [11]. After decapitation, brains were rapidly removed and placed in ice-cold artificial cerebrospinal fluid (ACSF; in mM: 126 NaCl, 2.5 KCl, 1 MgCl_2_, 1 CaCl_2_, 1.25 KH_2_PO_4_, 26 NaHCO_3_, and 20 glucoses, 290-300 mOsm, pH 7.4) oxygenated with 95% O_2_ and 5% CO_2_. Transverse hippocampal slices of 320 μm thickness were cut with a Leica VT1200s vibratome (Leica Biosystems, Wetzlar, Germany), then incubated with ACSF saturated with 95% O_2_ and 5% CO_2_ at 28°C for 1 h prior to the start of electrophysiological recordings.

fEPSPs were recorded with glass patch pipettes filled with ACSF positioned in the stratum radiatum of the CA1 subarea. A concentric electrode (CBARB75; FHC, Bowdoin, USA) was used to elicit synaptic responses by stimulating the Schaffer collateral fibers. Single baseline stimulation was delivered with an intensity that elicited ~50% of the maximum amplitude at 0.05 Hz. Long-term potentiation (LTP) was induced by two consecutive 1-s trains of 100-Hz stimuli, with a 20-s interval between trains. LTP was quantified by comparing the mean fEPSP slope during the baseline period with the mean fEPSP slope during the last 10 min of the recording period and calculating the percentage change from baseline. The paired-pulse ratio (PPR) of the fEPSP was defined as the percentage change in the amplitude of the second evoked response relative to the first, with a 50-ms interval between pulses. The intensity used for the PPR recordings was 0.2 mA. The input-output curves for fEPSP amplitude were recorded with gradually increasing intensities (0.1, 0.15, 0.2, 0.25, and 0.3 mA). All signals were amplified by an Axon-700B amplifier, filtered at 2 kHz, and sampled at 10 kHz with a Digidata 1440 device. Traces were acquired with Clampex 10.2 and analyzed in Clampfit 10.2 software.

### Golgi staining

Briefly, mice were decapitated, and the brains were immersed in a mixture of solutions 1 and 2 and kept in a dark container for 14 days. Then, the brains were transferred into solution 3 and stored in the dark for 3 days at 4°C. The brains were cut into 150-µm-thick sections at the location of the hippocampus with a vibratome and cryostat. The sections were stained according to the manufacturer’s instructions and then cover-slipped with neutral balsam mounting medium and observed under a light microscope with the help of an oil immersion objective [19]. Dendritic crossings in pyramidal neurons were analyzed with Sholl analysis. The count of dendritic intersections in the CA1 subfield included apical and basal dendrites. Neurons with mainly intact and fully impregnated apical and basal arborizations without truncated branches were analyzed. For analysis of dendritic interactions, 120-μm-long CA1 apical and basal dendritic sections from 14 to 16 randomly selected pyramidal cells from 4 mice were examined in Image J software. The number of spines was counted along a 50 μm linear length of an apical second-order branch.

### Morris water maze

The Morris water maze (MWM) test was conducted on 6 consecutive days to assess spatial memory acquisition and retention in mice [11]. The MWM consisted of a black round stainless steel tank (120 cm diameter) filled with water (30 cm deep, 22-26°C), Milk was added to the water to make it opaque. The maze video-tracking software (ANY-maze) divided the tank into four quadrants, and a platform (10 cm in diameter, 1 cm below the water surface) was placed in the center of one of the quadrants. Mice were required to find the hidden platform within 60 s (escape latency) by using environmental spatial clues and were allowed to stay on the platform for 20 s to learn its location. If the mouse failed to find the platform, it was guided to the platform and a score of 60 s was recorded as the escape latency. During the learning process, mice were subjected to four consecutive trials per day at 10 min intervals for 5 days, starting (facing the wall) from one of four points in the water maze. On the sixth day, the hidden platform was removed, and each animal was subjected to a probe trial (60 s). The time and swimming distance in the vicinity of the platform were recorded and analyzed.

### Novel object recognition

This experiment took place over two consecutive days [20]. On the first day, the mouse was placed in a 40 × 40 cm open arena for 5 min to acclimatize. The next day, two objects of the same shape and color were put into the same arena and then the mouse was returned to the arena for 5 min, and the total times the mouse spent exploring the two objects were recorded. 1 h later, one of the objects was replaced with a new one that differed both in shape and color, and the time the mouse spent exploring each object over 5 min was measured. Cognitive function was assessed by a discrimination index (DI), calculated as the time spent on each object (novel minus familiar/total object exploration). A positive number indicates a preference for the novel object (normal cognition) and a negative number indicates a preference for the familiar object (abnormal cognition).

**Figure 1.**
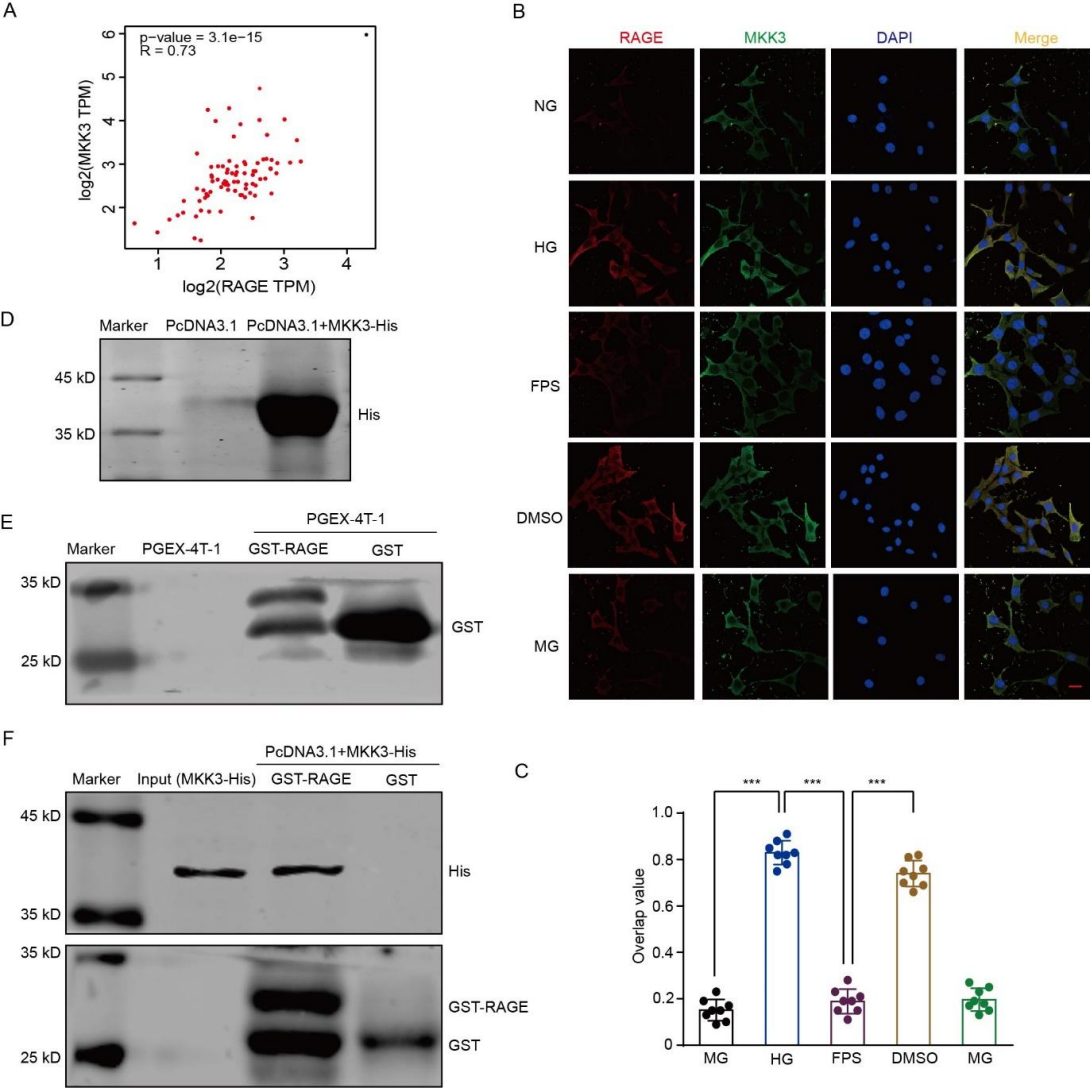
**MKK3 binds directly to RAGE**. NG: normal glucose group (25 mM glucose); HG: high-glucose group (50 mM glucose); FPS: RAGE-inhibitor group (high-glucose group with 2.5 μM FPS-ZM1 dissolved in DMSO); DMSO: solvent control group (high-glucose group with 2.5 μM DMSO); MG: mannitol control group (isosmotic pressure control, normal glucose group treated with 25 mM mannitol). (**A**) Analytical result from GEPIA 2 (cancer-pku.cn) showing a positive correlation between MKK3 and RAGE. (**B**) Representative laser scanning confocal microscopy images showing colocalization of RAGE and MKK3 in HT-22 cells in the high glucose condition. Red, RAGE; green, MKK3; blue, cellular nuclei labeled by DAPI. Colocalization of RAGE and MKK3 is shown in yellow. The scale bar is 10 μm (magnification ×400). (**C**) Overlap values showing RAGE and MKK3 colocalization. Overlap data were analyzed by one-way ANOVA followed by Tukey's test. F (4, 35) = 338.20. *** p < 0.001. n = 8 in each group. (**D and E**) Typical blots showing His-labeled pcDNA3.1-MKK3 and GST-labeled PGEX-4T-1-ct-RAGE after purification and overexpression. (**F**) Blots showing His-marked MKK3 pulled down by GST-tagged ct-RAGE.

### Fear conditioning

The fear conditioning test was a classic experiment that evaluates cognitive function [20]. The test was performed in a Plexiglas training chamber with a stainless-steel grid floor for shock delivery. The mouse was kept in the chamber and observed for 3 min. Three 30-s tone stimuli (2000 Hz, 70 dB) were presented with a 1-min interval, accompanied by a shock (0.7 mA) during the last 2 s of each tone. After three tone-shock pairings, the mouse was removed, and the chamber was cleaned with 95% ethyl alcohol for the next animal. The next day, the mouse was returned to the training chamber for 8 min with no tone and no foot shock, and the ratio of the freezing time was recorded as an index of contextual fear conditioning. Two hours later, the mouse was placed in a novel chamber for a 3-min adaptation period, and then the 2000 Hz, 70 db tone was transmitted continuously for 30 s without shock. The ratio of freezing time to the total 3 min was used as a measure of cued fear conditioning.

### Statistical analysis

All data were analyzed in GraphPad Prism 7.0 software. First, we evaluated the data for normality (Shapiro-Wilk test) and equal variance (F-test). When the data met the assumptions for parametric tests, Student’s t-tests were used to compare two groups, and one-way analyses of variance (ANOVAs) followed by Tukey post hoc tests were used for comparisons among multiple groups. Data from the MWM training sessions, the number of dendritic intersections, and fEPSCs were analyzed with two-way ANOVAs followed by Tukey’s multiple comparisons test. *p* < 0.05 was considered statistically significant. All data are shown as the mean ± standard error. Sample sizes are in line with previous studies in this field. Experimental animals were assigned to different groups randomly, and all investigators were blinded to the group allocations during the experiments and when assessing the outcomes.

## RESULTS

### MKK3 combines with ct-RAGE in a high-glucose environment

First, we evaluated whether the expression of MKK3 and RAGE were correlated using the “Correlation Analysis” tool of the Gene Expression Profiling Interactive Analysis (GEPIA) database. Fig. 1A shows that the protein expression of MKK3 and RAGE was positively correlated (*r* = 0.73). In addition, MKK3 immunofluorescence results showed that the colocalization of MKK3 with RAGE was higher in the high-glucose condition than in the normal-glucose condition; the RAGE inhibitor FPS-ZM1 blocked this increase but the FPS-ZM1 solvent control (DMSO alone) did not (*F*_(4, 35)_ = 338.20, all *p* < 0.001, Fig. 1B and C). Subsequently, His-tagged MKK3 protein and GST-tagged ct-RAGE (the intracellular segment of RAGE) were purified and overexpressed by Pet28a and PcDNA3.1 plasmids, respectively (Fig. 1D and E), and then we conducted a GST pull-down analysis to evaluate whether MKK3 binds to ct-RAGE. Consistent with the results from our previous study, we found that MKK3 binds ct-RAGE directly (Fig. 1F).

### MKK3 binds directly to RAGE AAs 362-365 through its K329 site

**Figure 2.**
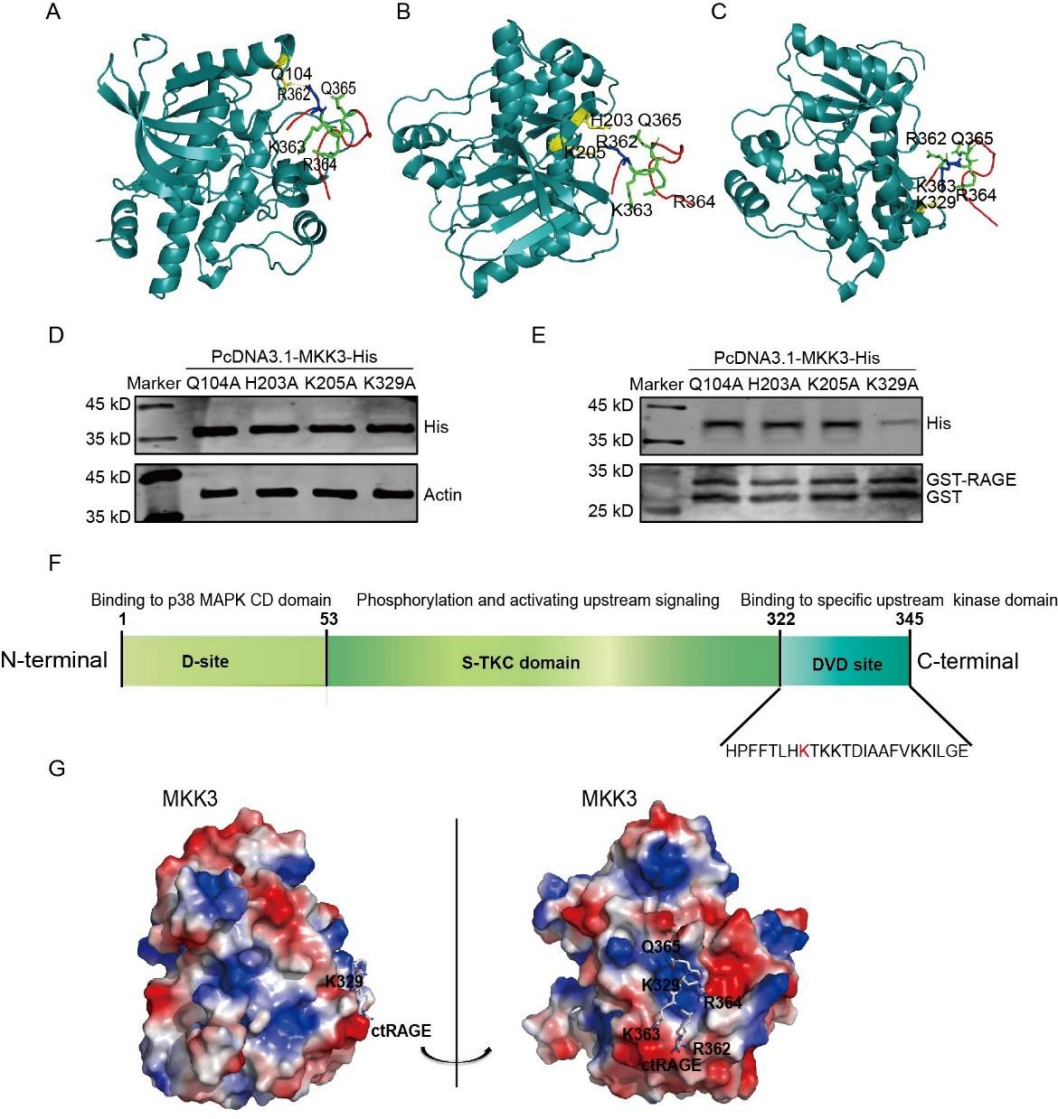
**K329 is the key site for MKK3 binding to RAGE**. (**A-C**) Cartoon linear docking figure showing possible sites of MKK3 binding to the cytoplasmic RKRQ motif in ct-RAGE. The cytoplasmic RKRQ motif are shown as sticks (colored by atom) and the related residues (Q104, H203, K205 and K329) in MKK3 are also shown as sticks. The polar binding sites between ct-RAGE and MKK3 are marked with yellow dotted lines. (**D**) Histagged site mutants (Q104A, H203A, K205A, and K329A) of MKK3 were packaged in PcDNA3.1 plasmid and overexpressed in HEK-293T cells, then tested by western blotting with anti-His. (**E**) K329A mutation specifically blocked the MKK3-RAGE interaction. GST-tagged ct-RAGE was expressed in BL21 cells and then subjected to GST pull-down analysis. MKK3 in the HEK-293T cells lysate was pulled down by GST-tagged ct-RAGE, but MKK3 with the K329A mutation could not bind to ct-RAGE. (**F**) Diagram showing the structure of MKK3. K329 is located in the DVD site. (**G**) Vacuum electrostatics map showing the spatial structure of ct-RAGE in combination with MKK3. In MKK3, positively charged surfaces are shown in blue, negatively charged surfaces are shown in red, and neutral surfaces are shown in white. K329 of MKK3 and AAs 362-365 of ct-RAGE are shown.

To determine the exact site by which MKK3 binds to ct-RAGE, we employed molecular docking to predict the possible spatial structure of MKK3 and RAGE AAs 362-365. We identified Q104, H203, K205, and K329 as probable sites of MKK3 binding to C-terminal RAGE as these are the sites involved in the polar contacts (Fig. 2A-C). We next constructed different MKK3 mutants (Q104A, H203A, K205A, and K329A) and used the PcDNA3.1 plasmid to transfect these into HEK-293 cells for overexpression (Fig. 2D). We next performed a GST pull-down analysis to determine the key site of MKK3 binding to RAGE. Fig. 2E shows that MKK3 with K329 mutation blocked the conjugation of MKK3 and RAGE, but the Q104A, H203A, and K205A mutants did not. Thus, MKK3 mutation at K329 was selected for the subsequent experiments.

It was reported that the DVD domain (AAs 322-345) is an important region for MKK3 binding to upstream proteins [21]. Consistently, MKK3 K329 is located in the DVD site (Fig. 2F). We also used a vacuum electrostatics map to examine the spatial details of MKK3 and ctRAGE binding. The vacuum electrostatics map identified K329 as the “central” site since it fits the spatial structure of ct-RAGE and multiple residues in the R362-K363-R364-Q365 motif (Fig. 2G). These results suggest that K329 is the key site through which MKK3 interacts directly with ct-RAGE.

### Mutating K329A disrupts the interaction between MKK3 and RAGE in HT-22 cells in the high-glucose condition

**Figure 3.**
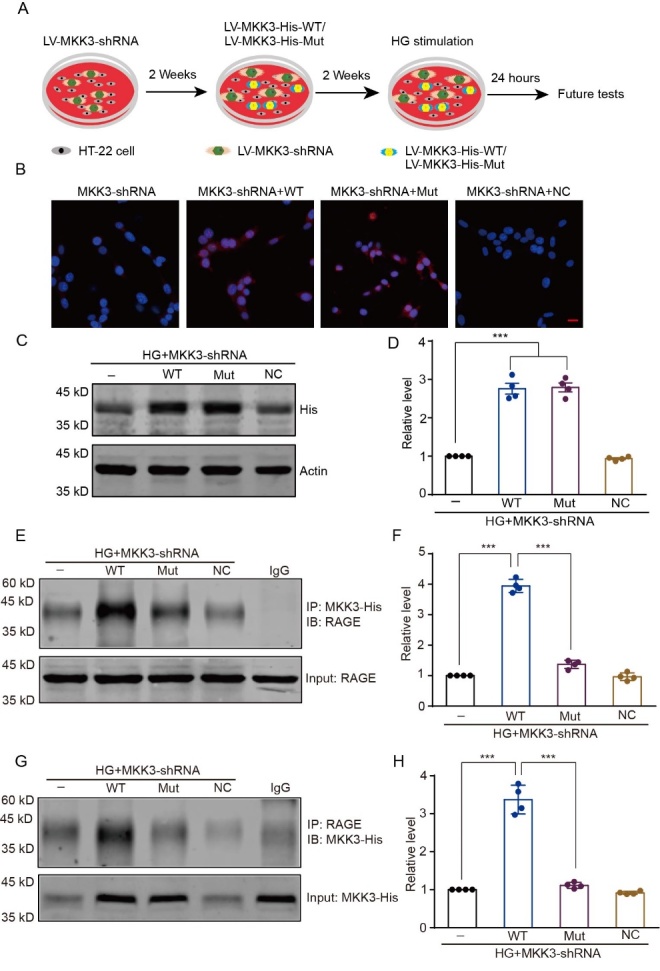
**Mutation of K329A disrupts the RAGE-MKK3 interaction in a high-glucose environment**. HG + LVMKK3-shRNA: MKK3-knockdown cells treated with high glucose; HG + LV-MKK3-shRNA + WT: HG + LVMKK3-shRNA cells with overexpression of wild-type MKK3; HG + LV-RAGE-shRNA + Mut: HG + LV-MKK3-shRNA cells with overexpression of the MKK3 K329 mutant; HG + LV-RAGE-shRNA + NC: HG + LV-MKK3-shRNA cells transfected with a nonsense control. (**A**) Schematic overview of the experimental design. (**B**) MKK3 wild-type and mutant were packaged in lentivirus and transfected into MKK3 knockdown HT-22 cells. (**C**) Expression of wild-type and mutant MKK3-His detected by western blotting with the His anti-body. (**D**) Optical density is presented as the fold change relative to the LV-MKK3-shRNA group. Data were assessed with a one-way ANOVA followed by Tukey's test. F (3, 12) = 129.00. *** p < 0.001. n = 4 in each group. (E and F) The combination of RAGE and His-MKK3 was detected by co-IP followed by western blotting with the His and RAGE antibodies respectively. (G and H) Optical density is shown as the fold change relative to the HG + MKK3-shRNA group. Data were analyzed with one-way ANOVAs followed by Tukey’s test. F (3, 12) =148.80 in D2 and 204.40 in E2. ***p < 0.001. n = 4 in each group.

Following our identification of the MKK3-RAGE binding site, we next investigated how mutating K329A affects the MKK3-RAGE interaction in a high-glucose environment. The experimental schedule is displayed in Fig. 3A. To avoid interference from endogenous MKK3, we first transfected LV-MKK3-shRNA into HT-22 cells. The data show that 4, 6, and 8 μL LV-MKK3-shRNA markedly decreased endogenous MKK3 expression (Supplementary Fig. 1A and B, *F*_(4, 15)_ = 154.00, all *p* < 0.001); we selected 4 μL LV-MKK3-shRNA for use in further experiments. Wild-type or mutant RFP-labeled LV-MKK3 was transfected into MKK3-knockdown HT-22 cells (Fig. 3B); a dosage of 2 μL led to significant overexpression of wild-type and mutant MKK3 and was selected for subsequent experiments (Supplementary Fig. 1 C-F, *F*_(4, 15)_ = 55.54 in B2 and 78.37, all *p* < 0.001; Fig. 3C and D, *F*_(3, 12)_ = 129.00, all *p* < 0.001). In MKK3-knockdown HT-22 cells, treatment with mutant MKK3 significantly inhibited MKK3 and RAGE conjugation after high-glucose stimulation, compared to treatment with wild-type MKK3 (Fig. 3E-H, *F*_(3, 12)_ = 148.80 in F and 204.40 in H, all *p* < 0.001). Therefore, MKK3 K329A mutation blocks the MKK3-RAGE interaction induced by high glucose.

### MKK3 mutation reduces inflammation in HT-22 cells by obstructing the RAGE-MKK3 interaction

**Figure 4.**
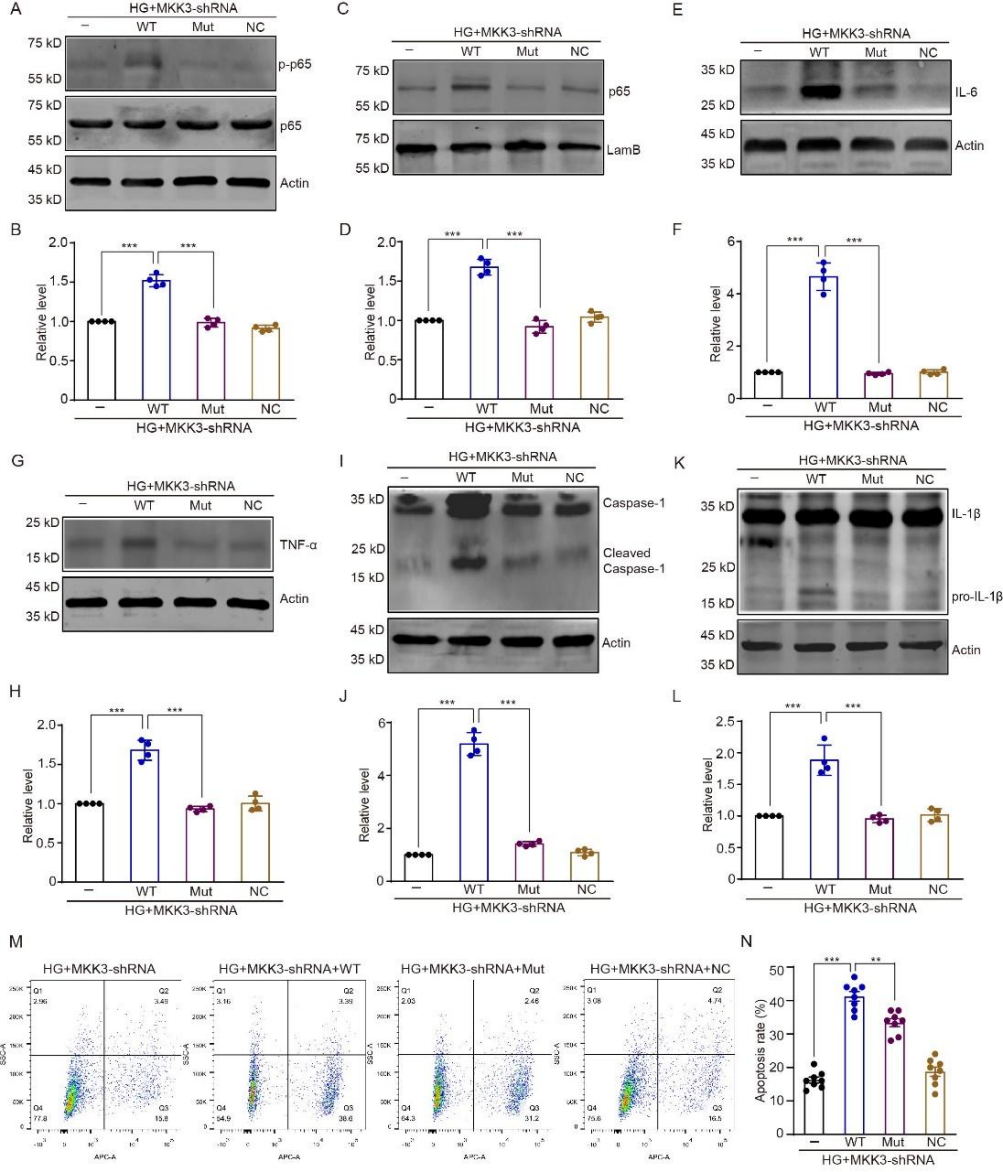
**MKK3 mutation decreases p65 nuclear transcription, TNF-α and IL-6 levels, and caspase-1 and IL-1β cleavage in HT-22 cells by disrupting the RAGE-MKK3 interaction**. (**A**) Phosphorylated p65 detected by Western blotting. (**B**) Relative intensity represented as the fold change relative to the HG + MKK3-shRNA group. Data were analyzed with a one-way ANOVA followed by Tukey’s test. F (3, 12) = 118.30. *** p < 0.001. n = 4 in each group. (C and D) Typical bands for p65 nuclear transcription and the relative intensity displayed as the fold change relative to the HG + MKK3-shRNA group. Data were analyzed with a one-way ANOVA followed by Tukey’s test. F (3, 12) = 93.32. *** p < 0.001. n = 4 in each group. (**E-L**) Expression of IL-6, TNF-α, cleaved caspase-1, and pro-IL-1β, and the relative intensity of these proteins presented as fold change relative to the HG + MKK3-shRNA group. Data were analyzed with one-way ANOVAs followed by Tukey’s test. F (3. 13) = 187.40 (C2), 74.16 (D2), 308.80 (E2) and 44.77 (F2) respectively. *** p < 0.001. n = 4 in each group. (**M**) Representative cellular flow cytometry images emerged cellular apoptosis. Dead cells in Q1, late apoptotic cells in Q2, viable apoptotic cells in Q3 and normal cells in Q4. (**N**) Apoptotic rate (number of cells in Q2 and Q3/total number of cells) were presented. One-way ANOVA followed by Tukey’s test was used. F(3, 28) = 102.50. *** p < 0.001; ** p = 0.003. n = 8 in each group.

NF-κB is a nuclear transcription factor whose phosphorylation and nuclear transcription contribute to the neuroinflammatory response, and subsequent neuronal injury, in a high-glucose environment [22]. In the present study, NF-κB showed the highest regulation score for MKK3 and RAGE (Supplementary Fig. 2A and B), as evaluated by the Cistrome DB Toolkit database (http://dbtoolkit.cistrome.org). We next ascertained whether and how K329A mutation mediates NF-κB pathway by blocking MKK3-RAGE interaction and exploring the downstream signaling pathway. Mutant MKK3 abrogated cytoplasmic p65 phosphorylation and reduced nuclear transcription of p65 and p50, but wild-type MKK3 did not (Fig. 4A-D, *F*_(3, 12)_ = 118.30 in B and 93.32 in D, all *p* < 0.001; Supplementary Fig. 3A and B, *F*_(3, 12)_ = 126.80, all *p* < 0.001).

We also measured inflammatory factors, such as interleukin-6 (IL-6), tumor necrosis factor α (TNF-α), cleaved caspase 1, and pro-interleukin-1β (pro-IL-1β), which are downstream signaling molecules of NF-κB. Immunoblotting analysis showed that these inflammatory factors were increased in MKK3-shRNA knockdown HT-22 cells that subsequently received high glucose and wild-type MKK3. Intriguingly, mutant MKK3 prevented these increases (Fig. 4E-L, *F*_(3. 13)_ = 187.40(F), 74.16(H), 308.80(J) and 44.77(L) respectively, all *p* < 0.001). Furthermore, we investigated the effects of NF-κB suppression on cell apoptosis. As shown in Fig. 4M and N, wild-type MKK3 significantly increased the apoptosis rate in HT22 cells with MKK3 knock-down (Fig. 4M and N, *F*_(3, 28)_ = 102.50, *p* < 0.001). Inhibition the canonical NF-κB pathway through K329A mutation protected the HT22 cells from high-glucose (Fig. 4M and N, *F*_(3, 28)_ = 102.50, *p* = 0.003). Taken together, these results show that MKK3 K329A mutation attenuates NF-κB nuclear transcription in canonical pathway and the expression of inflammatory factors, and that this protective role occurs because the mutation blocks the interaction between MKK3 and RAGE.

**Figure 5.**
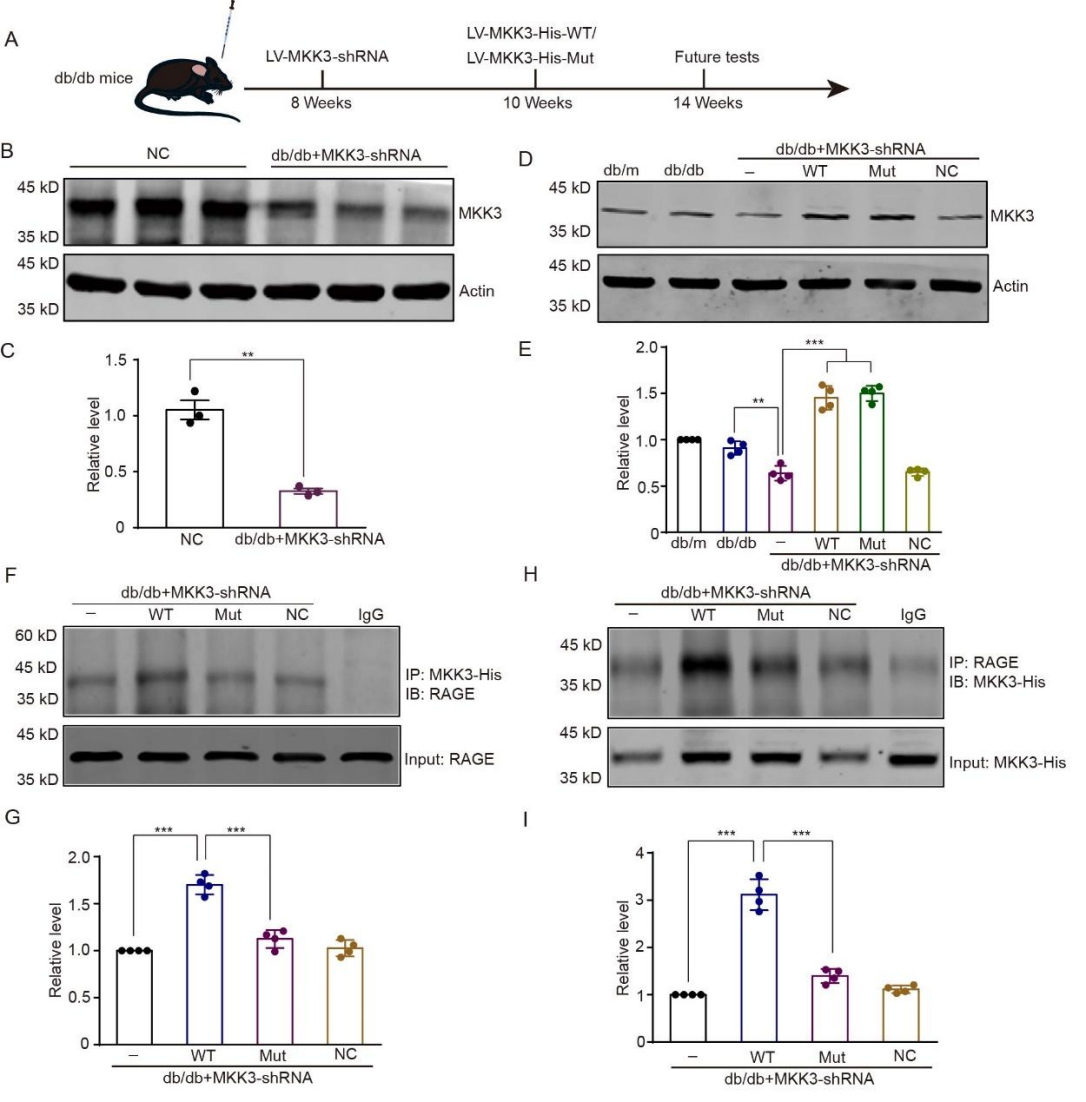
**K329A mutation reduces the RAGE-MKK3 interaction in the hippocampus of db/db mice**. db/m: ageand sex-matched normoglycemic heterozygous littermates; db/db: diabetic model group; db/db + LV-MKK3-shRNA: db/db male mice that received LV-MKK3-shRNA; db/db + LV-MKK3-shRNA + WT: MKK3-knockdown db/db mice injected with wild-type MKK3; db/db + LV-MKK3-shRNA + Mut: MKK3-knockdown db/db mice injected with mutant MKK3; db/db + LV-MKK3-shRNA + NC: MKK3-knockdown db/db mice injected with nonsense control. (**A**) Schematic of the experimental design. (B and C) Inhibition of MKK3 was assessed by Western blotting; fold change is relative to the NC group. Data were analyzed with t tests, ** p = 0.002. (D and E) The level of wild-type and mutant MKK3 detected by western blotting; fold change is relative to the db/m group. Data were assessed with a one-way ANOVA followed by Tukey’s test. F (8, 18) = 93.04. ** p = 0.002; *** p < 0.001. n = 4 in each group. (F and G) Co-precipitation of RAGE with wild-type or mutant MKK3 was detected by co-IP followed by western blotting with RAGE and His antibodies respectively. (H and I) Optical density presented as the fold change relative to the db/db + MKK3-shRNA group. Results were analyzed with one-way ANOVAs followed by Tukey’s test. F (3, 12) = 63.64 (D2) and 115.30 (E2). *** p < 0.001. n = 4 in each group.

### MKK3 mutation disrupts the interaction between MKK3 and RAGE in the hippocampus of db/db mice

Given the above findings, we next used db/db mice as a model of diabetes to test the effect of MKK3 mutation in vivo. We focused on the hippocampus because it is a key brain region in the regulation of cognition and is highly susceptible to hyperglycemia [23]. To suppress endogenous MKK3, LV-MMK3-shRNA was microinjected bilaterally into hippocampal CA1. Two weeks later, EGFP-tagged LV-MKK3-His-Wt or -Mut under the neuronal CAP-Syn promoter was injected bilaterally into the hippocampus to overexpress wild-type or mutant MKK3 in hippocampal neurons (Fig. 5A).

LV-MKK3-shRNA reduced the MKK3 level in the hippocampus of db/db mice (Fig. 5B and C, *p* = 0.002). The subsequent injections of wild-type and mutated LV-MKK3 significantly elevated the MKK3 level (Fig. 5D and E, *F*_(8, 18)_ = 93.04, both *p* < 0.001.). An immunofluorescence assay also showed wild-type and mutated LV-MKK3 expressed in hippocampus (Supplementary Fig. 4A). Consistent with the results in HT-22 cells, the conjunction of MKK3 and RAGE in the hippocampus was significantly decreased with injection of mutant MKK3 compared with wild-type MKK3 (Fig. 5F-I, *F*_(3, 12)_ = 63.64(D2) and 115.30(E2), all *p* < 0.001). These results confirm that K329 is a key site for the binding of MKK3 to RAGE in a hyperglycemic environment.

**Figure 6.**
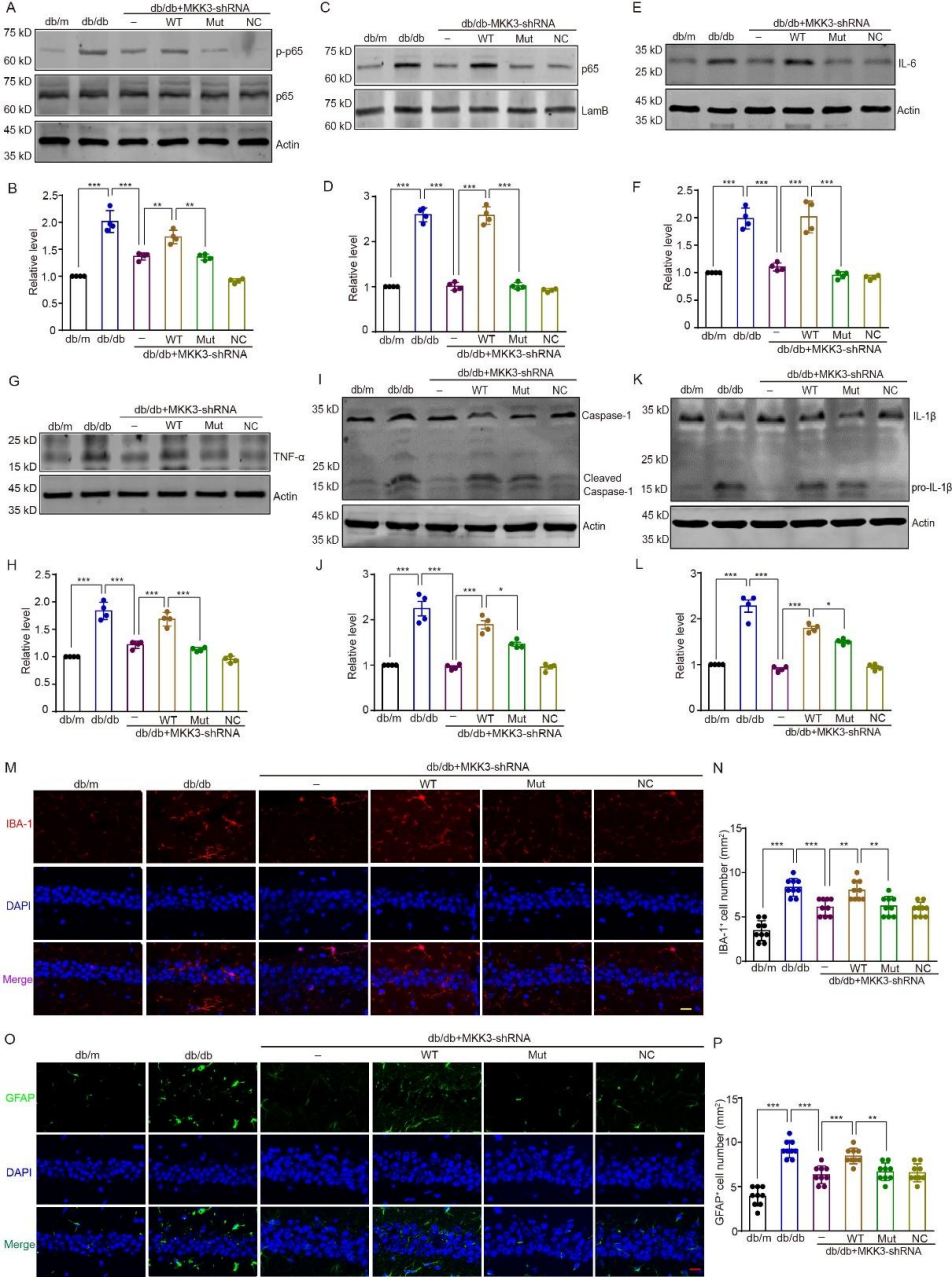
**K329A mutation ameliorates neuroinflammation in the hippocampus by disrupting the interaction of MKK3 and RAGE**. (A and B) Level of p-p65 assessed by western blotting with anti-p-65 antibody; optical density is shown as the fold change relative to the db/m group. Data were analyzed with a one-way ANOVA followed by Tukey’s test. F (5, 18) = 64.54. ** p = 0.002 (db/db + MKK3-shRNA + WT group vs db/db + MKK3-shRNA group); ** p = 0.001 (db/db + MKK3-shRNA + Mut group vs db/db + MKK3-shRNA + WT group); *** p < 0.001. n = 4 in each group. (C and D) Protein level of p65 assessed by western blotting; fold change is relative to the db/m group. Data were analyzed by one-way ANOVA followed by Tukey’s test. F (5, 18) = 218.70. *** p < 0.001. n = 4 in each group. (**E-H**) IL-6 and TNF-α expression levels assessed by Western blotting Optical density is presented as the fold change relative to the db/m group. Results were analyzed with one-way ANOVAs followed by Tukey’s test. F (5, 18) = 50.10 and 65.76 respectively. *** p < 0.001. n = 4 in each group. (**I-L**) Expression of cleaved caspase-1 and IL-1β; the fold change is relative to the db/m group. Data were analyzed with one-way ANOVAs followed with Tukey’s test. F (5, 18) = 47.87 and 81.79 respectively. *** p < 0.001; * p = 0.023 (E2) and 0.041 (F2). n = 4 in each group. (**M**) Microglia activation in the hippocampus detected by immunofluorescence with anti-IBA-1 antibody. IBA-1 is shown in red and cellular nuclei (DAPI) are shown in blue. IBA-1+ cells were considered to be activated microglia. Scale bar is 20 μm (magnification ×400). (**N**) Number of IBA-1+ microglia per 1-mm length. n = 4 in each group (two or three brain slices per mouse). Data were analyzed with a one-way ANOVA followed by Tukey’s test. F (5, 48) = 26.82. *** p < 0.001; ** p = 0.004 (db/db + MKK3-shRNA + WT group vs db/db + MKK3-shRNA group) and 0.007 ((db/db + MKK3-shRNA + Mut group vs db/db + MKK3-shRNA + WT group). (**O**) Representative images of astrocyte activation in the hippocampus. GFAP is shown in green and cellular nuclei (DAPI) are shown in blue. GFAP+ cells were considered to be activated astrocytes. Scale bar is 20 μm (magnification ×400). (**P**) Number of GFAP+ astrocytes per 1-mm length, n = 4 in each group (two or three brain slices per mouse). Data were assessed with a one-way ANOVA followed by Tukey’s test. F (5, 48) = 31.92. *** p < 0.001; ** p = 0.005.

### Mutation of MKK3 protects against hippocampal neuroinflammation in db/db mice by disrupting the MKK3-RAGE interaction

Next, we explored the influence of MKK3 K329A on neuroinflammation induced by hyperglycemia. Fig. 6A and B show that the level of phosphorylated p65 in the hippocampus of db/db mice was higher than in db/m mice *(F*_(5, 18)_ = 64.54, *p* < 0.001) and that knockdown of MKK3 significantly reduced this increase (*p* < 0.001). Subsequent overexpression of wild-type MKK3 enhanced p65 phosphorylation (*p* = 0.002), but mutant MKK3 did not. Furthermore, K329 mutation significantly inhibited p65 and p50 nuclear transcription in db/db mice with MKK3 knockdown, whereas wild-type MKK3 did not have this effect (Fig. 6C and D, *F*_(5, 18)_ = 218.70, both *p* < 0.001; Supplementary Fig. 5A and B, *F*_(5, 18)_ = 184.50, both *p* < 0.001).

Inflammatory factors, such as IL-6, TNF-α, cleaved caspase-1, and pro-IL-1β are downstream of the NF-κB signaling pathway and are also involved in neuroinflammation induced by hyperglycemia [24,25]. We therefore asked whether mutating MKK3 affects the levels of these inflammatory factors in the hippocampus. As shown in Fig. 6E-L, the expression levels of IL-6, TNF-α, cleaved caspase-1, and pro-IL-1β were significantly higher in the HG+MKK3-shRNA+WT group than in the HG+MKK3-shRNA group, but this increase was blocked by mutation of MKK3 (*F*_(3. 13)_ = 187.4(F), 74.16(H), 308.80(J) and 44.77(L) respectively, all *p* < 0.001).

Next, we performed IBA-1 and GFAP immunofluorescence to investigate the effect of MKK3 mutation on the activation of microglia and astrocytes in the hippocampus of db/db mice. CA1 is a major subregion of the hippocampus involved in cognition and memory [26,27]. The number of IBA-1^+^ puncta was significantly increased in db/db mice compared with db/m mice (Fig. 6 M and N, *F*_(5, 48)_ = 26.82, *p* < 0.001), but this increase was prevented by knockdown of MKK3 (*p* < 0.001). Subsequent overexpression of wildtype MKK3 significantly boosted IBA-1 expression in MKK3 knockdown db/db mice (*p* = 0.004) but mutant MKK3 did not (*p* = 0.004). Similarly, the number of GFAP^+^ astrocytes was significantly higher in db/db mice than in db/m mice, and overexpression of wild-type MKK3 increased the number of GFAP^+^ astrocytes in MKK3 knockdown db/db mice (Fig. 6O and P, *F*_(5, 48)_ = 31.92, *p* < 0.001). As with IBA-1, overexpression of mutant MKK3 resulted in reduced astrocyte activation than overexpression of wild-type MKK3 (*p* = 0.005). Taken together, these results indicate that MKK3 K329A mutation attenuates hippocampal neuroinflammation in db/db mice by interrupting the interaction between MKK3 and RAGE and thus inhibiting the canonical NF-κB signaling pathway.

### MKK3 mutation ameliorates synaptic dysfunction in the hippocampus

**Fig 7.**
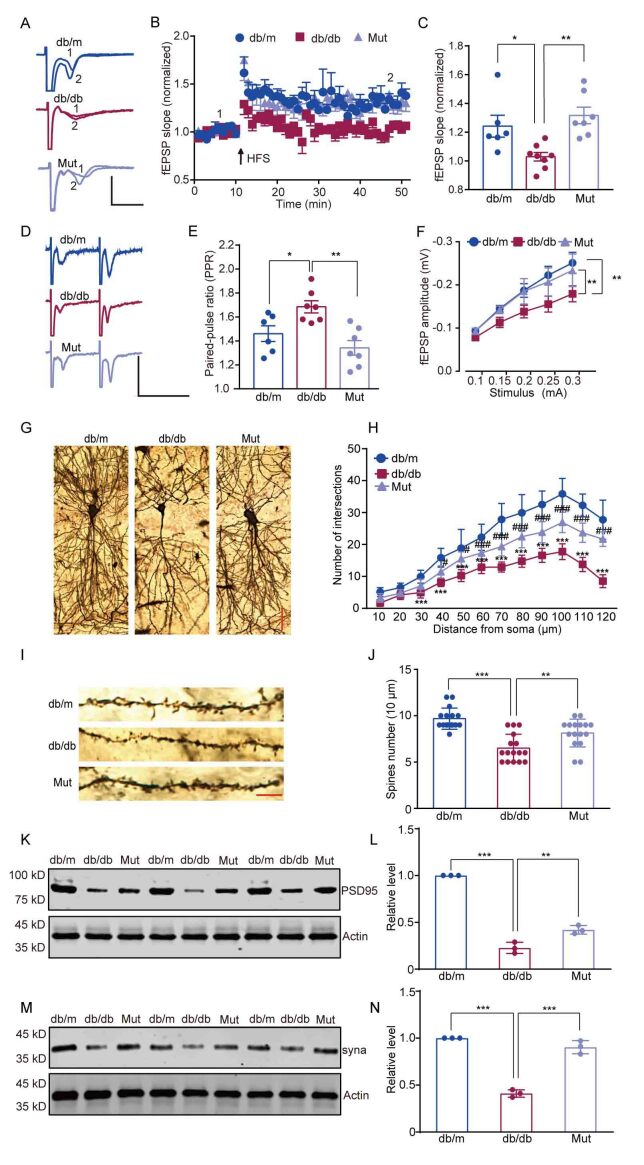
**MKK3 mutation ameliorates synaptic dysfunction in db/db mice**. db/m: age- and sex-matched normoglycemic heterozygous littermates; db/db: diabetic model group; Mut: MKK3-knockdown db/db mice injected with mutant MKK3. (A and B) Representative traces and time-course of fEPSP slopes during LTP recordings in the hippocampus. Scale bars represent 0.5 mV and 10 ms. (**C**) MKK mutation rescued the LTP fEPSP deficit in db/db mice. The relative increase in fEPSPs during the last 10 min of recording was analyzed with a one-way ANOVA followed by Tukey’s test. n = 3 (two or three brain slices per mouse). F (2, 17) = 14.94. * p = 0.032, ** p = 0.003. (**D**) Representative paired-pulse traces. Scale bars represent 0.5 mV and 50 ms. (**E**) MKK3 mutation rescued the change in PPR in db/db mice. Data were analyzed with a one-way ANOVA followed by Tukey’s test. F (2, 17) = 9.13. * p = 0.042, ** p = 0.002. n = 3 (Two or three brain slices per mouse). (**F**) MKK3 mutation rescued the deficit in fEPSP amplitude in db/db mice. Data were analyzed by two-way ANOVA followed by Tukey’s test. q (60, 155) = 5.032 (db/db group vs db/m group) and 4.219 (Mut group vs db/db group). ** p = 0.004 (db/db group vs db/m group) and 0.001 (Mut group vs db/db group) respectively. n = 4 (three or four brain slices per mouse). (**G**) Dendritic branches in hippocampal neurons were detected by Golgi staining. Scale bar is 50 μm (magnification ×200). (**H**) Thenumber of dendritic intersections was analyzed by Sholl analysis. Data were analyzed with a two-way repeated-measures ANOVA followed by Tukey's test. n = 4 (three brain slices per mouse). 30 μm to 120 μm from the soma, db/db compared with db/m, *** p < 0.001. 40 and 50 μm from the soma, Mut group compared with db/db, * p = 0.025 and 0.021 respectively. 60 μm to 120 μm from the soma, Mut group compared with db/db, *** p < 0.001. (**I**) Representative images showing dendritic spine density and morphology. Scale bar is 10 μm (magnification ×600). (**J**) Dendritic spine number was counted in Image J, and data were analyzed with a one-way ANOVA followed by Tukey's test. F (2, 45) = 20.98. *** p < 0.001; ** p = 0.005. n = 4 (three or four sections per mouse). (K and L) Immunoblotting analysis of PSD95 levels in the hippocampus of db/db mice in which endogenous MKK3 was knocked down and replaced with mutated MKK3. Fold change is relative to the db/m group, and data were analyzed with a one-way ANOVA followed by Tukey’s test. *** P < 0.001; ** P = 0.004. F (2, 6) = 254.30. (M and N) Representative bands for synaptophysin; fold change is relative to the db/m group. Data were analyzed with a one-way ANOVA followed by Tukey’s test. F (2, 6) = 137.90. *** p < 0.001. n = 4 in each group.

MKK3 and the activation of downstream signaling factors are required for synaptic dysfunction and abnormalities in diabetes and neurodegenerative diseases [10,11,28]. We, therefore, tested the impact of specific mutation of MKK3 on the maintenance of LTP, a form of synaptic plasticity that is widely thought to be the basis of learning and memory in db/db mice. Electrophysiological recordings in hippocampal slices showed that high-frequency stimulation (HFS)-induced LTP of field excitatory postsynaptic potentials (fEPSPs) was significantly inhibited in db/db mice, but not in db/db mice injected with mutated MKK3 (Fig. 7A-C, *F*_(2, 17)_ = 14.94, *p* = 0.032 and 0.003 respectively). To determine whether there were presynaptic changes in the CA1 subregion of the hippocampus, we measured the paired-pulse ratio (PPR) of the fEPSPs. The PPR with a 50-ms interval showed a significant increase in db/db mice, but MKK3 mutation rescued this effect (Fig. 7D and E, *F*_(2, 17)_ = 9.13, *p* = 0.042 and 0.002 respectively). To examine the effect of a specific mutation of MKK on synaptic transmission, we recorded the fEPSP input-output curves. The input-output curves were reduced in db/db mice compared with db/m mice, but mutation of K329A in MKK markedly improved synaptic transmission in db/db mice (Fig. 7F, q_(60, 155)_ = 5.032 and 4.219, *p* = 0.004 and 0.001 respectively). These findings demonstrate that MKK mutation alleviates impairments in synaptic function in db/db mice.

**Figure 8.**
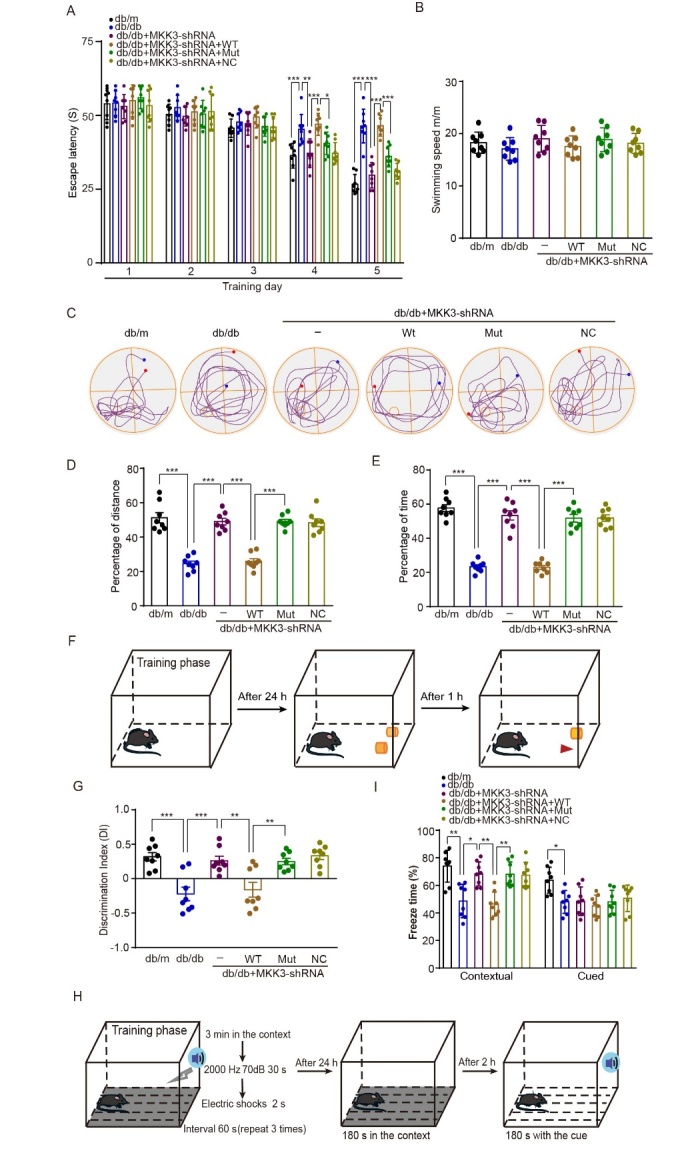
**MKK3 mutation ameliorates cognitive impairments in db/db mice**. (**A**) Average escape latency of mice to reach the platform on five consecutive training days in the Morris water maze. Data were analyzed with a two-way ANOVA and Tukey's multiple comparisons test, n = 8 in each group. On the fourth day, *** p < 0.001; ** p = 0.002; * p = 0.028. q (8, 210) = 6.05 (db/db group vs db/m group), 5.50 (db/db + MKK3-shRNA group vs db/db group), 6.64 (db/db + MKK3-shRNA + Wt group vs db/db + MKK3-shRNA group), 4.36 (db/db + MKK3-shRNA + Mut group vs db/db + MKK3-shRNA + Wt group). On the fifth day, *** p < 0.001. q (8, 210) = 13.37 (db/db group vs db/m group), 11.30 (db/db + MKK3-shRNA group vs db/db group), 11.34 (db/db + MKK3-shRNA + Wt group vs db/db + MKK3-shRNA group), 7.03 (db/db + MKK3-shRNA + Mut group vs db/db + MKK3-shRNA + Wt group). (**B**) Swimming speed during the probe trial (no platform) on the sixth day. Data were analyzed with a one-way ANOVA and Tukey’s test. n = 8 in each group. *** p < 0.001. (**C**) Track maps of the probe trial on the sixth day (the red dot indicates the start, and the blue dot indicates the end). (D and E) Percentage of distance and time spent in the target quadrant during the probe trial (n = 8 mice per group). Data were analyzed with one-way ANOVAs followed by Tukey’s test. F (5, 42) = 39.21 in A4 and 67.33 in A5. *** p < 0.001. (**F**) Schematic of the novel object recognition task. (**G**) Discrimination index. n = 8 in each group. Results were analyzed with a one-way ANOVA followed by Tukey’s test. F (5, 42) = 4.08. *** p < 0.001; ** p = 0.004 (db/db + MKK3-shRNA + Wt group vs db/db + MKK3-shRNA group) and 0.005 (db/db + MKK3-shRNA + Mut group vs db/db + MKK3-shRNA + Wt group). (**H**) Schematic of the fear conditioning test. (**I**) Percentage of time spent freezing in the contextual and cued fear conditioning trials. Data were analyzed with one-way ANOVAs followed by Tukey's test. n = 8 in each group. F (4, 35) = 13.02 for contextual fear conditioning, whereas F (4, 35) = 9.27 for cued fear conditioning. In the contextual fear conditioning, ** p = 0.003 (db/db vs db/m), 0.002 (db/db + MKK3-shRNA + Wt group vs db/db + MKK3-shRNA group) and 0.002 (db/db + MKK3-shRNA + Mut group vs db/db + MKK3-shRNA + Wt group); * p = 0.016 (db/db + MKK3-shRNA vs db/db group). In the cued fear conditioning, * p = 0.037 (db/db vs db/m).

Following on from our observation that mutated MKK3 improves synaptic functioning in diabetic mice, we evaluated synaptic morphology in the CA1 subregion using Golgi staining. We observed that both the apical and basal dendrites of hippocampal pyramidal neurons had significantly fewer intersections in db/db mice than in db/m mice, but this hyperglycemia-induced reduction was partially reversed in db/db mice with MKK3 knockdown and subsequent overexpression of the MKK3 K329 mutant (Fig. 7G and H, both *p* < 0.001). In addition, there were significantly fewer dendritic spines in db/db mice than in db/m mice (Fig. 7I and J, *F*_(2, 45)_ = 20.98, *p* < 0.001), but the K329 mutation similarly reversed this effect (*F*_(2, 45)_ = 20.98, *p* = 0.005). To verify the effects of MKK3 mutation on synapse-related proteins, we examined the expression of PSD-95 and synaptophysin (Fig. 7K-N). PSD-95 and synaptophysin expression levels were reduced in the hippocampus of db/db mice (*F*_(2, 6)_ = 254.30 in L and 137.90 in N, both *p* < 0.001), and MKK3 mutation ameliorated these decreases (*p* = 0.004 in L and *p* < 0.001 in N). Thus, mutating MKK3 at K329 contributes to the restoration of synaptic form and structure in the hippocampus of db/db mice.

**Figure 9.**
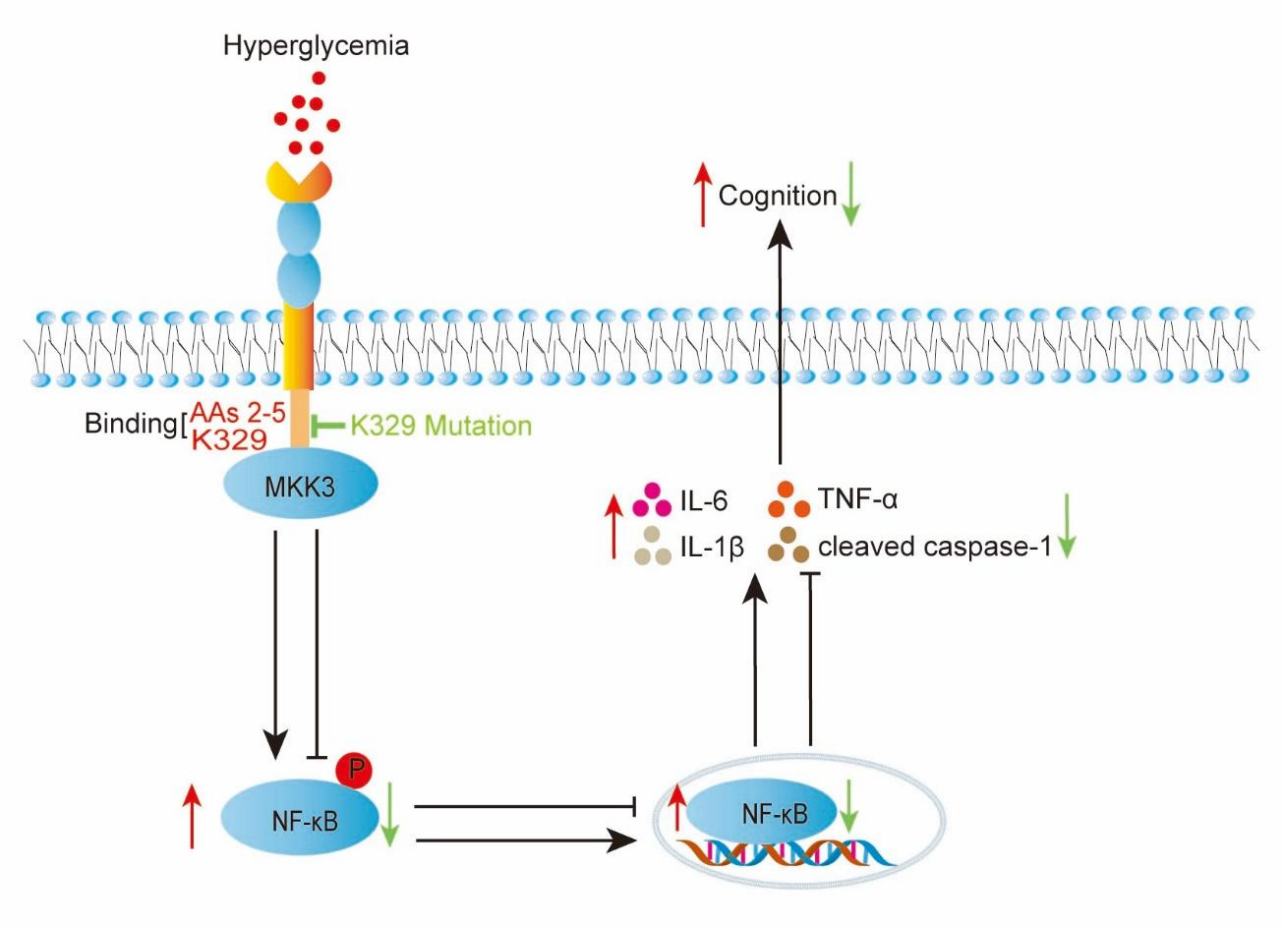
**Graphical abstract illustrating the mechanism by which the MKK3-RAGE interaction regulates cognition in db/db mice**. Direct binding of MKK3 K329 to ct-RAGE AAs 2-5 facilitates neuroinflammation, causing cognitive decline. Mutation of K329 ameliorates the cognitive decline by blocking the interaction between MKK3 and RAGE.

### MKK3 mutation reverses cognitive impairments in db/db mice

Synaptic loss and decreased synaptic plasticity are associated with structural and functional abnormalities in the hippocampus, and these changes are believed to be the basis of the cognitive deficits in diabetes [29]. To explore whether mutating MKK3 reverses the learning and memory impairments caused by diabetes, we tested mice with the Morris water maze (MWM), the novel object recognition (NOR) test, and a fear conditioning test. In the MWM, the escape latency for mice in all six groups did not differ over the first three days of training (Fig. 8A). On the fourth and fifth days, the db/db mice had a prolonged escape latency compared with the db/m mice (Fig. 8A, q_(8, 210)_ = 6.05 and 13.37, both *p* < 0.001). As expected, knock down of MKK3 significantly decreased the escape latency in db/db mice (*p* = 0.002 on the fourth day and *p* < 0.001 on the fifth day). MKK3 knockdown db/db mice that received wild-type MKK3 took more time to find the platform on the fourth and fifth days (q_(8, 210)_ = 6.64 and 11.34, both *p* < 0.001). However, MKK3 knockdown mice that received mutant MKK3 found the platform in a significantly shorter time (q_(8, 210)_ = 4.36 on the fourth day and 11.03 on the fifth day, *p* = 0.028 and *p* < 0.001 respectively). There were no significant differences in swimming speed between the six groups during the day 6 probe trial (Fig. 8B). Sample probe-trial swimming paths of mice in each group are displayed in Fig. 8C. In the probe trial, db/db mice swam for less time and a shorter distance in the target quadrant, but MKK3 knockdown reversed this. MKK3 knockdown mice with overexpression of mutant MKK3 swam for significantly more time and a longer distance in the target quadrant than mice with overexpression of wild-type MKK3 (Fig. 8D and E, *F*_(5, 42)_ = 39.21 in D and 67.33 in E, all *p* < 0.001).

A schematic of the NOR test is shown in Fig. 8F. There was a decrease in the discrimination index (DI) in db/db mice, indicating cognitive impairment, but this decline was not evident in db/db mice with MKK3 knockdown (Fig. 8G, *F*_(5, 42)_ = 4.08, both *p* < 0.001). Similar to the results for the MWM, the DI in knockdown mice treated with mutant MKK3 was higher than in knockdown mice treated with wild-type MKK3 (*p* = 0.004). Fig. 8H shows the experimental procedure for the fear conditioning test. As expected, freezing time was reduced in db/db mice compared with db/m mice in both the contextual and cued fear conditions (Fig. 8I, *F*_(4, 35)_ = 13.02 in contextual fear conditioning, *p* = 0.003, while *F*_(4, 35)_ = 9.27 in cued fear conditioning, *p* = 0.037). MKK3 knockdown mice froze more than db/db mice in the contextual fear condition trial (*p* = 0.016). Freezing time was significantly longer in db/db knockdown mice treated with mutant MKK3 than knockdown mice treated with wild-type MKK3 in the contextual fear condition test (*p* = 0.002). Notably, there were no significant differences in freezing between db/db mice and db/db mice receiving different treatments in the cued fear-conditioning test. Overall, our results indicate that MKK3 mutation ameliorates the cognitive deficits caused by diabetes by blocking the binding of MKK3 to RAGE.

## DISCUSSION

In this comprehensive study, we report that the MKK3-RAGE interaction plays a complex role in diabetes-induced cognitive disorder and explore potential mechanisms of this role in vitro and in vivo. To our knowledge, our study provides the first evidence that the K329 site of MKK3 binds to the C-terminal of RAGE can promote neuroinflammation and synaptic dysfunction, thereby reducing cognitive function. We demonstrated that MKK3 mutation ameliorated DACD, reduced neuroinflammation, and restored synaptic function in the hippocampus of the db/db mice by disrupting the interaction of MKK3 and RAGE (Fig. 9). Collectively, these data strongly support MKK3 as a novel drug target for treating DACD.

Clinically, individuals with diabetes often exhibit cognitive dysfunction, characterized by reductions in executive functioning, rapid processing of information, and psychomotor and cognitive efficiency, which ultimately lead to a reduced quality of life [30,31]. Diabetes-induced brain hyperglycemia and glucose metabolic disorders are closely associated with the accumulation of AGEs, formed by glucose and ribose, which can accelerate DACD pathogenesis [32]. AGEs do not directly damage neural structure and function, but have their effect via the C-terminal of RAGE, which triggers downstream intracellular signaling pathways [33]. Consistent with this, we previously reported that RAGE contributes to neuronopathies and cognitive dysfunction by binding of C-terminal AAs 2-5 to MKK3 and subsequent activation of the p38MAPK signaling pathway [11]. However, the MKK3 sites responsible for binding to RAGE remained unknown. In the present study, we demonstrated that K329 of MKK3 is the crucial site for MKK3-RAGE conjunction under high glucose conditions.

MAPK cascades regulate multiple cellular events, ranging from cellular growth to apoptosis, in response to extracellular stimuli [34,35]. A conserved docking site, termed DVD, is a stretch of about 20 amino acids close to the C-terminal side of the MAPK catalytic domain, which directly binds to a specific upstream signaling protein to activate the downstream signaling pathway. There are three major MAPK subfamilies, namely MKK1, MKK4/7, and MKK3/6 [21]. MKK1 is typically involved in regulating growth and developmental signaling, MKK4/7 regulates apoptosis and many other disease-relevant processes, and MKK3/6 is involved in the regulation of inflammation [10,36-38]. Of MKK3 and MKK6, MKK3 is the most attractive target for genetic deletion because it is nonredundant in some pathological processes, such as those associated with obesity, hyperglycemia, and neuroinflammation [28,39]. Consistent with this, MKK3 is an important intracellular binding protein for RAGE in the hippocampus of db/db mice. Strikingly, we found that the K329 site located in the DVD domain was critical for this interaction and for the downstream effects on the pathology of DACD.

Growing evidence suggests that neuroinflammation plays a critical role in the development of cognitive deficits caused by diabetes [40,41]. Hyperglycemia-induced production of pro-inflammatory cytokines from activated microglia and reactive astrocytes could directly contributes to neuronal dysfunction, including impairment of synaptic function, loss of dendritic complexity, reduced number of synapses, decreased plasticity, etc. which hurts neuronal function and behavior [42,43]. The canonical NF-κB signaling pathway downstream of MAPK is the most prevalent pro-inflammatory mediator and is activated in microglia, astrocytes, and neurons in a high-glucose environment [44]. NF-κB exists as a heterodimer composed of p50 and the active RelA/p65 subunit, which are sequestered in the cytoplasm by binding with an inhibitor of κB (IκB) [45]. Mechanistically, MKK3 activation induces IκB-α dephosphorylation and departure from NF-κB, which readily translocates into the nucleus where it controls the transcription of multiple genes responsible for inflammation [46,47]. Recent studies have demonstrated that hyperglycemia gives rise to NF-κB transcription, with consequent negative impacts on synaptic plasticity, learning, and memory [24]. In the present study, we observed that hyperglycemia induces microglial and astrocytic activation and causes neuronal NF-κB transcription, which in turn activates downstream pro-inflammatory factors that leads to neuroinflammation. Therefore, our results suggest that NF-κB activity may contribute to DACD through activation of microglia and astrocytes and subsequent secretion of pro-inflammatory factors.

Previous studies have emphasized that interactions between proteins form the pathophysiological basis of many neurological and neurodegenerative diseases [48]. Protein complexes accumulate in response to various pathogen-associated molecular patterns and damaged-associated molecular patterns, but this ultimately results in cellular toxicity and metabolic disorders in the brain [49]. Blocking the protein binding sites by mutation or with specific interfering peptides can either inhibit the formation of protein complexes or decrease downstream signaling of the assembly [50,51]. We discovered that mutation of the K329 residue prevents MKK3 binding to RAGE, reducing activity in the canonical NF-κB signaling pathway in the hippocampus of db/db mice and thereby inhibiting neuroinflammation, improving synaptic structure and function, and ultimately attenuating DACD.

The hippocampus is a key brain region in the regulation of cognition and emotion and exhibits early vulnerability during the progression of DACD [52]. Inflammation in the hippocampus, particularly in the CA1 subfield, can impair neuronal structure and function and has been implicated in the pathogenesis of neurodegenerative diseases [26,27]. We have demonstrated in this study that diabetes-induced dysfunction in hippocampus-dependent learning and memory is associated with neuroinflammation caused by activation of the NF-κB signaling pathway. Previous studies found that hyperglycemia has a detrimental effect on the dendritic spines of pyramidal neurons in the hippocampus, with decreases in both the number of spines and synaptic plasticity [53,54]. Furthermore, hippocampal synapse-associated proteins, including synaptophysin and PSD95, are suppressed in diabetes [55]. Consistent with these results, our study confirmed that db/db mice have significant dysfunction in synaptic structure and plasticity in the hippocampus. However, replacing endogenous MKK3 with mutated MKK3 mitigated these synaptic impairments, reversed the decrease in synaptic proteins, and improved cognitive function in db/db mice. Therefore, attenuation of neuroinflammation and consequent reversal of synaptic changes in the hippocampus could be an important therapeutic strategy for DACD.

### Limitations

It is imperative to acknowledge that the development of DACD involves a multifaceted pathophysiological process that encompasses various factors, including glucose and lipid metabolism disorders, mitochondrial dysfunction, neuroinflammation, and impaired glutamic acid-glutamine cycle [56,57]. Although there may be other pathways and proteins that contribute to DACD, in-depth research is required to explore them fully. Furthermore, the definite molecular and cellular mechanism of RAGE and MKK3 interaction specifically affect the NF-κB signaling pathway, and what downstream events are responsible for the DACD will be studied in our further research. The MKK3-RAGE combination may probably lead to DACD through other signaling pathways. Noticeably, RAGE-targeted therapies have not yet been used in clinical trials or approved for treating DACD. However, we firmly believe that further basic and clinical trials are necessary to support the translation of these findings from mouse model to human. Consequently, more research in these fields needs to be undertaken to enhance the understanding of the complexity of this disease and improve its treatment.

### Conclusion

In conclusion, our work identifies the key site through which MKK3 binds to RAGE in the cytoplasm and the molecular mechanism by which the MKK3-RAGE interaction induces DACD. Furthermore, we showed that mutating MKK3 and thus blocking the MKK3-RAGE interaction, have a protective effect against diabetes-induced cognitive impairment by alleviating neuroinflammation and restoring function at hippocampal synapses. Understanding the binding mechanism between MKK3 and RAGE may be useful for the development of inhibitors targeting MKK3 and RAGE, providing novel insights into treating diabetes-associated neurodegenerative diseases.

## Supplementary Materials

The Supplementary data can be found online at: www.aginganddisease.org/EN/10.14336/AD.2024.0222.

### Supplementary Materials


