# Supplementary material for: MKK3 K329 Mutation Attenuates Diabetes-Associated Cognitive Dysfunction by Blocking the MKK3-RAGE Interaction and Inhibiting Neuroinflammation
Source: Aging Dis. 2024 Feb 22;16(1):598–618. doi: 10.14336/AD.2024.0222 (PMC11745445; doi:10.14336/AD.2024.0222)
Supplement: Supplementary file 1 [file AD-16-1-598-s.pdf]

SUPPLEMENTARY DATA

MKK3 K329 Mutation Attenuates Diabetes-Associated Cognitive Dysfunction by Blocking the MKK3–RAGE Interaction and Inhibiting Neuroinflammation

Changjiang Ying, Yan Li, Shidi Wu, Lin Gao, Yandong Zhu, Ye Qian, Xiangru Wen, Hui Li, Chengyu Huang, Bin Hu, Yuanjian Song, Xiaoyan Zhou

© 2024. Ying C et al. Published online at http://www.aginganddisease.org/EN/10.14336/AD.2024.0222

SUPPLEMENTARY DATA

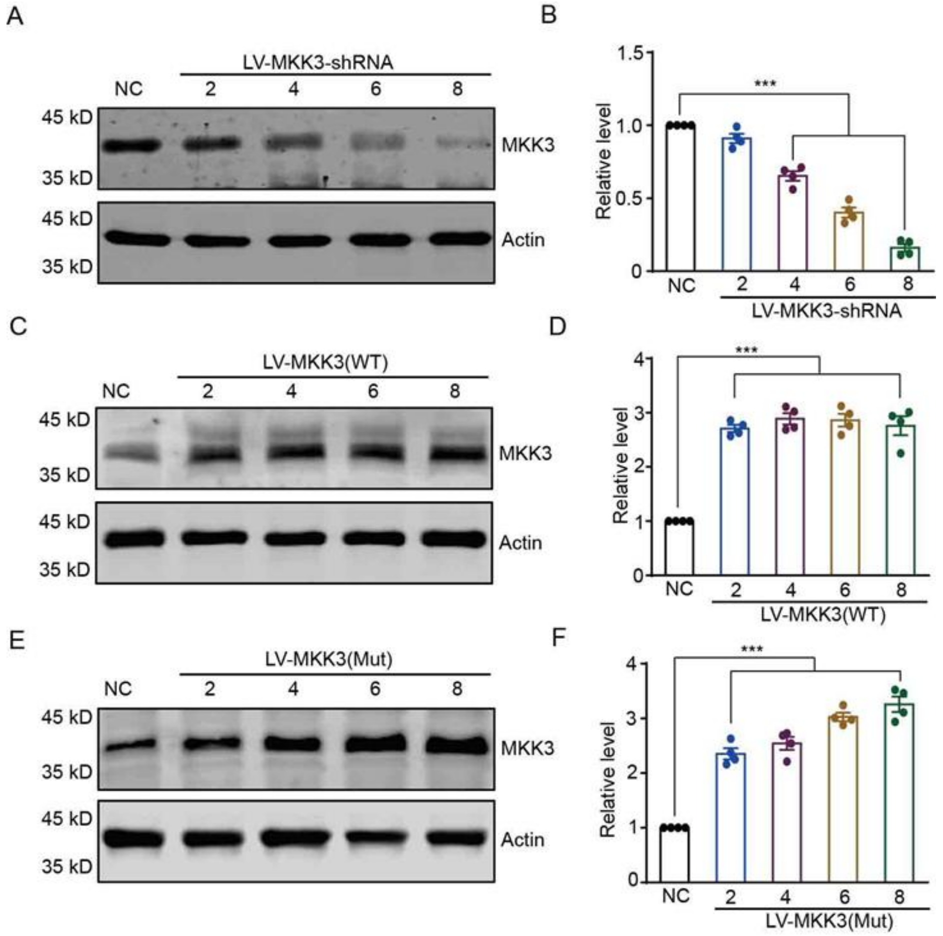

Supplementary Figure 1. MKK3-specific knockdown and overexpression in HT-22 cells. (A and B) Representative blots showing MKK3 knockdown with different doses of LV-MKK3-shRNA (2, 4, 6, and 8 μL; virus titration: 10^8 TU/mL). Relative level of MKK3 displayed as fold change relative to the NC group. Data were analyzed with one-way ANOVA followed by Tukey's test. $F_{(4, 15)} = 154.00$. *** $p < 0.001$. n = 4. **(C and D)** Different doses of wild-type MKK3 (virus titration: 10^8 TU/mL) were transfected into MKK3 knockdown HT-22 cells; optical density is shown as the fold change relative to the NC group. Data were analyzed with a one-way ANOVA and Tukey's test. $F_{(4, 15)} = 55.54$. *** $p < 0.001$. n = 4. **(E and F)** Different doses of mutant MKK3 (virus titration: 10^8 TU/mL) were transfected into MKK3 knockdown HT-22 cells; optical density is shown as the fold change relative to the NC group. Results were analyzed with one-way ANOVAs followed by Tukey's test. $F_{(4, 15)} = 78.37$. *** $p < 0.001$. n = 4 in each group.

© 2024. Ying C et al. Published online at http://www.aginganddisease.org/EN/10.14336/AD.2024.0222

SUPPLEMENTARY DATA

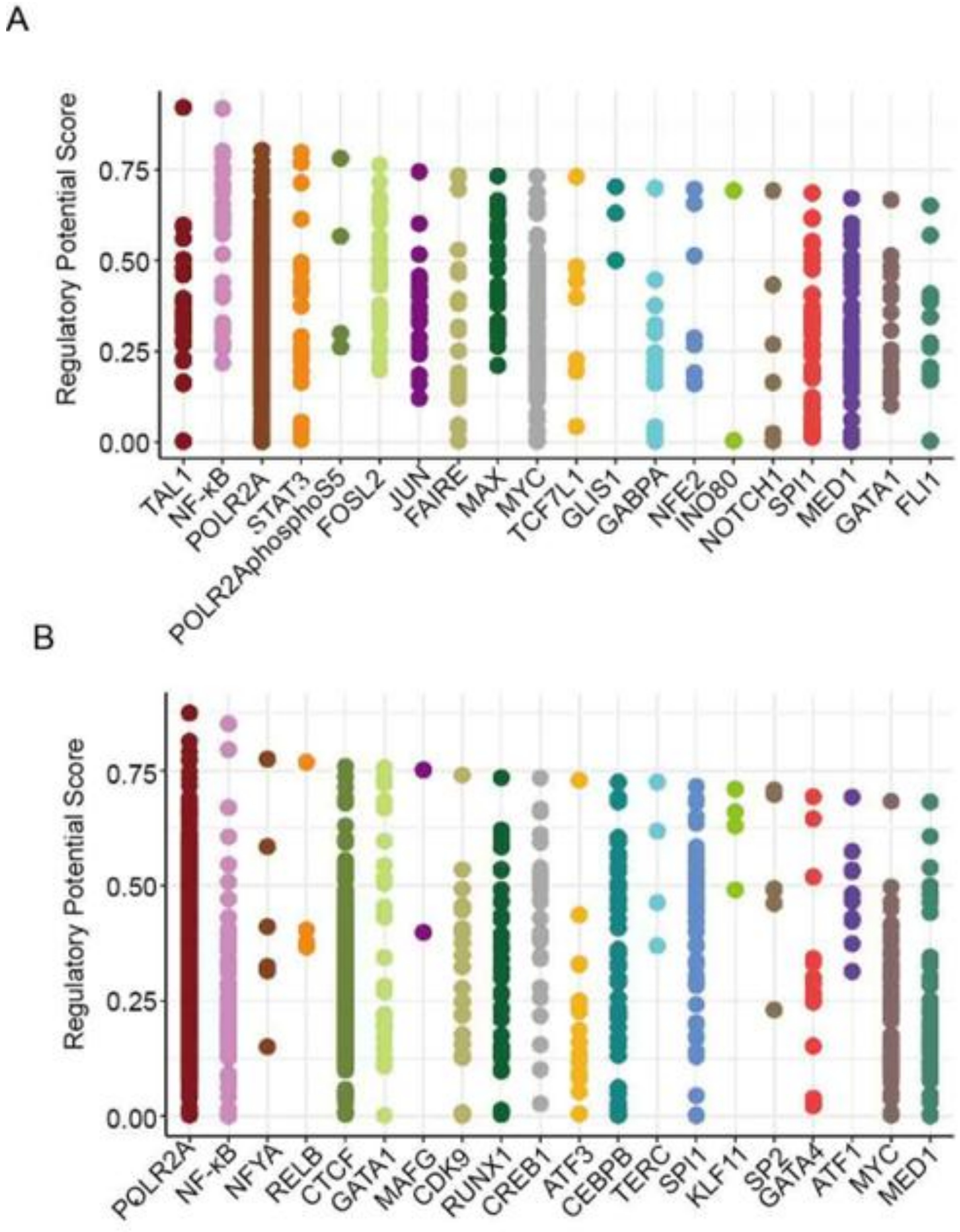

Supplementary Figure 2. MKK3–RAGE interaction regulates the NF-κB signaling pathway. (A and B) Regulatory potential scores for regulatory factors for MKK3 and RAGE, respectively, from the Cistrome DB Toolkit database. NF-κB is common to both MKK3 and RAGE.

© 2024. Ying C et al. Published online at http://www.aginganddisease.org/EN/10.14336/AD.2024.0222

SUPPLEMENTARY DATA

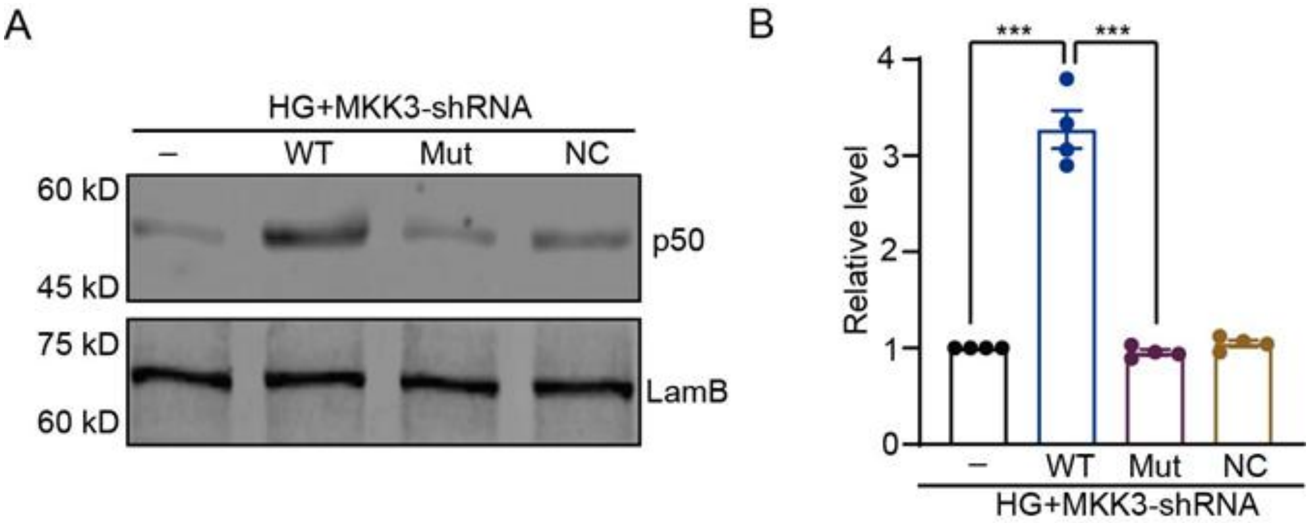

Supplementary Figure 3. K329 mutation inhibits p50 nuclear transcription by blocking the RAGE–MKK3 interaction. (A) Phosphorylated p50 was tested by western blotting. **(B)** Relative intensity represented as the fold change relative to the LV-MKK3-shRNA group. Data were analyzed with one-way ANOVA followed by Tukey's test. $F_{(3, 12)} = 126.80$. *** $p < 0.001$. n = 4 in each group.

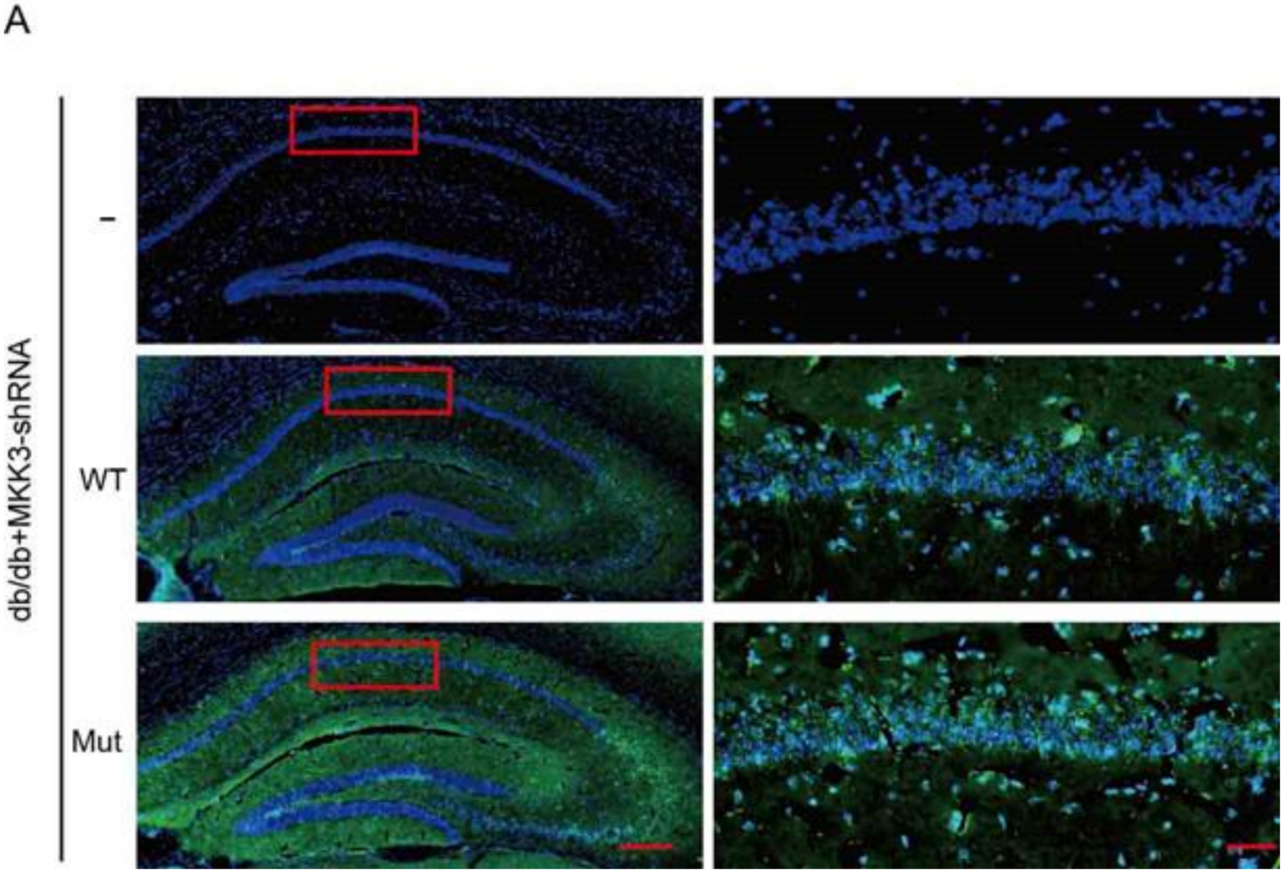

Supplementary Figure 4. Overexpression of EGFP-tagged MKK3-shRNA-WT/Mut in hippocampal sections. (A) Typical fluorescence figures showing overexpression of GFP-labeled wild-type and mutant LV-MKK3 in the hippocampal CA1 subregion. The red boxed areas in the left column were revealed at higher magnification in the right column respectively.Left: Scale bar = 100 µm (40× magnification); Right: Scale bar = 20 µm (400× magnification).

© 2024. Ying C et al. Published online at http://www.aginganddisease.org/EN/10.14336/AD.2024.0222

SUPPLEMENTARY DATA

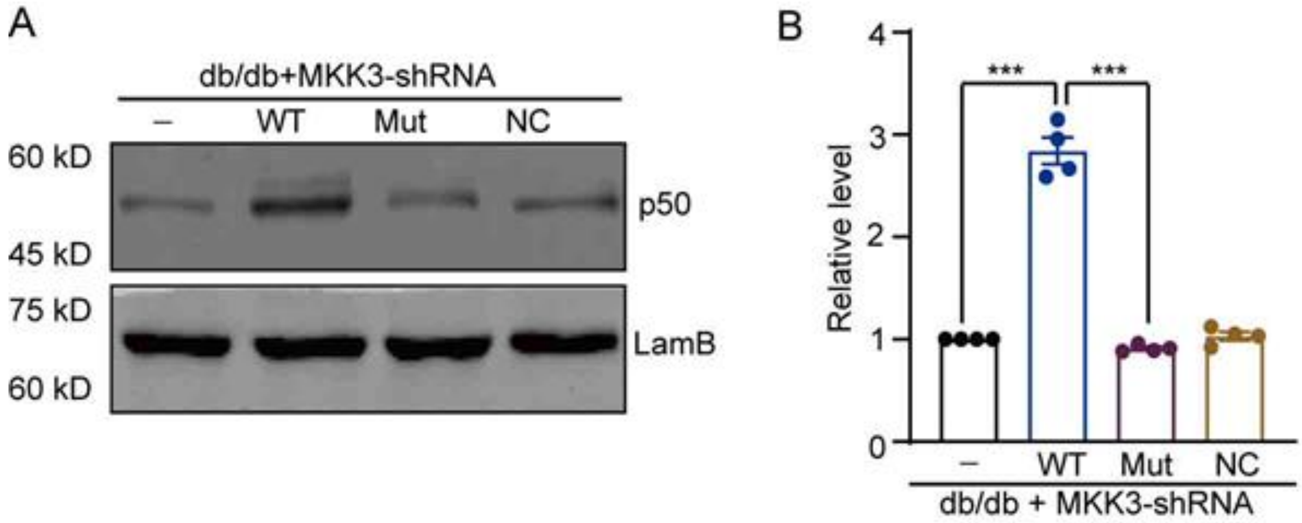

Supplementary Figure 5. Mutational MKK3 decreases the level of p50 in nucleus. (A) Level of p50 assessed by western blotting with anti-p50 antibody. **(B)** optical density is displayed as the fold change relative to the db/db + MKK3-shRNA group. Data were analyzed with one-way ANOVA followed by Tukey's test. $F_{(5, 18)} = 184.50$. *** $p < 0.001$. n = 4 in each group.

Supplementary Table 1. All chemicals, recombinant proteins, critical commercial assays, cell lines, experimental models, oligonucleotides, and recombinant DNA used in the present work.

Chemicals		
FPS-ZM1	MedChemExpress	945714-67-0
TransInTM EL Transfection Reagent	Beijing TransGen Biotech	FT201-01
protein A/G-Agarose	MedChemExpress	HY-K0202
IPTG	VICMED	367-93-1
Puromycin	VICMED	58-58-2
G-418	VICMED	108321-42-2
DMEM High Glucose	KeyGEN BioTECH	KGM1280
QuickBlock Western	Beyotime	P0252
Immobilon-NC transfer membrane	MERCK	HATF00010
Antibody diluent	VICMED	VP6022

Critical Commercial Assays		
Enhanced BCA Protein Assay Kit	Beyotime	P0010S
FD Rapid GolgiStain Kit	Fdneurotech	PK401
Nucleoprotein Extraction Kit	Keygentec	KGP150
PierceTM GST Protein Interaction Pull-Down Kit	Thermo Fisher Scientific	TJ2276008
Plasmid microextraction Assay Kit	Tiangen Biotech	DP106
Annexin V Apoptosis Assays Kit	Keygentec	KGA1021

Cell Lines	
HEK-293T	Gift from Xuzhou Medical University Public Experimental Research Center
HT-22	Gift from Xuzhou Medical University Public Experimental Research Center

Experimental Models: Organisms/Strains	
Mouse: C57BL/6	Xuzhou Medical University Laboratory Animal Center
Mouse: db/db and db/m	Model Animal Research Center of Nanjing University

Oligonucleotides	
MKK3shRNA:	SANGON

© 2024. Ying C et al. Published online at http://www.aginganddisease.org/EN/10.14336/AD.2024.0222

SUPPLEMENTARY DATA

CCGGCCCATTCTTCACCTTGCACAACTCGAGTTGTGCAAGGTGAAGAATGGGTTTTT<br>NC：<br>GTTCTCCGAACGTGTCACGTCAAGAGATTACGTGACACGTTCGGAGAATT	SANGON

Recombinant DNA and Recombinant Protein	
Plasmid PGEX-4T-1-GST-RAGE 362-365(RKRQ)	SANGON
Plasmid PGEX-4T-1-GST	SANGON
Plasmid PcDNA3.1-His-MKK3	SANGON
Plasmid pcDNA3.1-His-MKK3 Q104A/H203A/K205A/K329A	SANGON
Plasmid pcDNA3.1-His	SANGON
Lentivirus hU6-MCS-CMV-Puromycin-MKK3-shRNA	GeneChem
Lentivirus hU6-MCS-CMV-Puromycin-NC	GeneChem
Lentivirus Ubi-MCS-RFP-3His-SV40-Neomycin-MKK3	GeneChem
Lentivirus Ubi-MCS-RFP-3His-SV40-Neomycin-MKK3 K329A	GeneChem
Lentivirus Ubi-MCS-EGFP-3His-SV40-Neomycin-MKK3	GeneChem
Lentivirus Ubi-MCS-EGFP-3His-SV40-Neomycin-MKK3 K329A	GeneChem

© 2024. Ying C et al. Published online at http://www.aginganddisease.org/EN/10.14336/AD.2024.0222
